# Correlative Photoactivated Localization and Scanning Electron Microscopy

**DOI:** 10.1371/journal.pone.0077209

**Published:** 2013-10-25

**Authors:** Benjamin G. Kopek, Gleb Shtengel, Jonathan B. Grimm, David A. Clayton, Harald F. Hess

**Affiliations:** Janelia Farm Research Campus, Howard Hughes Medical Institute, Ashburn, Virginia, United States of America; Julius-Maximilians-University Würzburg, Germany

## Abstract

The ability to localize proteins precisely within subcellular space is crucial to understanding the functioning of biological systems. Recently, we described a protocol that correlates a precise map of fluorescent fusion proteins localized using three-dimensional super-resolution optical microscopy with the fine ultrastructural context of three-dimensional electron micrographs. While it achieved the difficult simultaneous objectives of high photoactivated fluorophore preservation and ultrastructure preservation, it required a super-resolution optical and specialized electron microscope that is not available to many researchers. We present here a faster and more practical protocol with the advantage of a simpler two-dimensional optical (Photoactivated Localization Microscopy (PALM)) and scanning electron microscope (SEM) system that retains the often mutually exclusive attributes of fluorophore preservation and ultrastructure preservation. As before, cryosections were prepared using the Tokuyasu protocol, but the staining protocol was modified to be amenable for use in a standard SEM without the need for focused ion beam ablation. We show the versatility of this technique by labeling different cellular compartments and structures including mitochondrial nucleoids, peroxisomes, and the nuclear lamina. We also demonstrate simultaneous two-color PALM imaging with correlated electron micrographs. Lastly, this technique can be used with small-molecule dyes as demonstrated with actin labeling using phalloidin conjugated to a caged dye. By retaining the dense protein labeling expected for super-resolution microscopy combined with ultrastructural preservation, simplifying the tools required for correlative microscopy, and expanding the number of useful labels we expect this method to be accessible and valuable to a wide variety of researchers.

## Introduction

Understanding cellular structure and the placement of molecular components within the cell is key to understanding cellular function. Since the advent of microscopes, biologists have probed cell structure and organization using various labeling technologies and stains. Many protocols have been developed for the localization of cellular components including immunolabeling with antibodies and fluorescent protein fusion [1]. However, all of the protocols have limitations including, but not limited to the fraction of the target protein labeled, specificity, resolution, and ultrastructural preservation.

Immunolabeling electron microscopy (EM) with a gold conjugated antibody (immunogold) is often the method of choice for precise, high-resolution localization of cellular components. While immunogold EM will remain a valuable tool for the foreseeable future, it has several notable drawbacks that have driven the development of alternative protocols. Foremost, it is often difficult or impossible to label a target protein with sufficient reliability and specificity to gain viable insight. Even under the best conditions, only a miniscule fraction of the target protein is likely to be successfully detected and provide a high confidence in the localization. This requires a highly-selective antibody with a high affinity for the object of interest and furthermore a balance of fixation and staining methods that provide optimal preservation of cellular ultrastructure while retaining both the accessibility and antigenicity of the antigen [2]. Many alternative immunogold EM protocols have been developed, however, high-quality immunogold EM remains challenging and requires often time-consuming protocol optimization.

An alternative for the localization of cellular components is correlative light and electron microscopy (CLEM) [3]. In CLEM, a fluorescent signal marks the location of an area of interest, such as an HIV particle [4], [5], that can then be correlated to an electron micrograph of the structure from which the fluorescence signal emanated. The variety of fluorescent proteins and dyes and attachment strategies vastly expand the protocol options and also enable live cells to be imaged prior to fixation. The main advantage of fluorescent fusion proteins is that they provide the highest specificity of any labeling method since the label and target are directly connected. One potential disadvantage of fluorescent protein fusion is that fusion may disrupt the functionality and/or localization of the wild-type protein target. Another disadvantage of fluorescence microscopy is that diffraction-limited optical imaging makes it impossible to precisely localize fluorescent protein signals within electron micrographs, in most cases limiting CLEM to identification and illustration purposes.

A further advantage of CLEM is conferred when one is able to combine super-resolution fluorescence microscopy with electron microscopy. Super-resolution fluorescence microscopy methods, such as PALM, provide nanometer localization precision of targets with the specificity of protein fusion [6]. Methods to combine super-resolution fluorescence microscopy (including PALM, stimulated emission depletion (STED), and stochastic optical reconstruction microscopy (STORM)) with electron microscopy have been developed by ourselves and others [6]–[8]. A major obstacle to achieving high-quality CLEM is balancing fluorophore and ultrastructural preservation. Invariably, either the strong fixation destroys antigenicity or the fluorophore itself leaving too few localization events and marginal fluorescent images, or a weaker fixation, while giving more localization events for better fluorescent images, fails to preserve the ultrastructure expected from EM images. Our previous correlative method involved three-dimensional (3D) imaging for both PALM and electron microscopy and achieved both excellent fluorophore and ultrastructural preservation [9]. However, the equipment required to perform the 3D correlative procedure is not commercially available or easily accessible. We modified this protocol to be compatible with two-dimensional (2D) imaging systems for super-resolution fluorescence microscopy, such as PALM, which is commercially available, and compatible with standard SEMs that have good low energy performance and are common in most research institutions.

In addition to redesigning a protocol to be compatible with a simpler imaging system, we wanted to show the versatility of this technique through multicolor imaging and the use of fluorescent dyes. For many experiments the localization of a single target is sufficient, but more often a cellular structure contains many components that need to be localized relative to each other or a structure of interest. Several studies have demonstrated simultaneous imaging of multiple fluorescent protein fusions with PALM microscopy [10], often using the second color as a spatial reference [11], [12]. Correlating the fluorescent protein signal to electron micrographs eliminates the need to use a second fusion protein as a spatial reference, thus opening up the second color for the imaging of another object of interest. There are certain instances when small-molecule dyes are advantageous to fluorescent protein fusions, even if they have less specificity, because they are less likely to disrupt protein function and/or localization and can be used as an alternative label or an additional color. Recently developed caged dyes show great promise as alternatives to fluorescent protein fusions for PALM [13], [14].

In this work, we describe a method to correlate super-resolution fluorescence images from PALM to SEM data that takes advantage of a simpler imaging system while still maintaining excellent fluorophore preservation and detailed electron microscopy ultrastructure. We demonstrate the versatility of this technique by imaging diverse cellular structures, and the ability to perform multicolor PALM imaging using two different fluorescent labels correlated to an SEM image. Lastly, we expand the labeling technologies that can be used with this method by using a small-molecule caged dye. All of these advances will serve to make correlative PALM/SEM a valuable tool for cell biologists.

## Results

### 2D Correlative PALM/SEM uses simpler imaging systems

We focused previously on 3D correlative microscopy using interferometric PALM (iPALM) [15] and focused ion beam ablation (FIB)-SEM to develop a method that can access the full structure of 3D objects [9]. However, 2D analysis is informative for many biological problems and, in general, the tools and equipment for 2D imaging are more readily available, less expensive, faster, and easier to use. Therefore, we wanted to perform correlative microscopy in a way that was amenable for 2D analyses. A major challenge we faced when trying to apply our correlative method [9] to 2D imaging was that the sections were covered in a layer of methylcellulose, which is necessary to relieve drying artifacts after staining [16]. The methylcellulose layer was not a problem when using FIB-SEM since this layer is cut away during imaging. However, the methylcellulose layer posed a problem for SEM imaging without FIB because only a minimal thickness of material over the specimen can be tolerated to allow the 1–2 KeV imaging electrons to penetrate the specimen. To overcome these issues, we adopted a cryosection positive staining protocol that also replaces methylcellulose with polyvinyl alcohol (PVA) [17].

**Figure 1:**
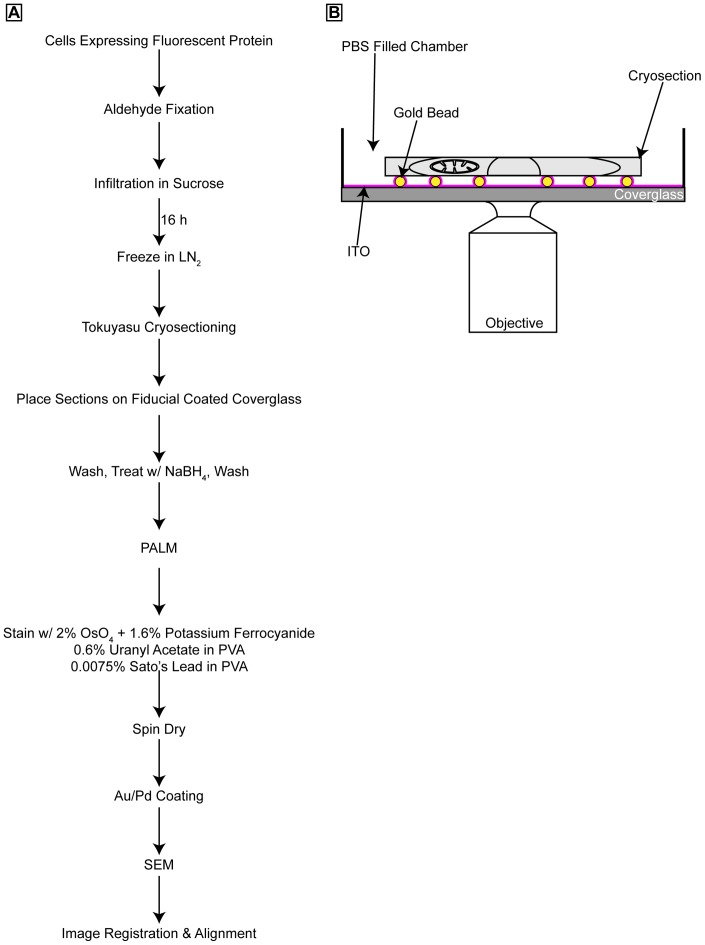
Overview of correlative PALM and SEM procedure and the PALM imaging system. (**A**) Flow diagram showing the steps involved in correlating PALM and SEM images. (**B**) PALM imaging set-up showing the 100 nm thick cryosection on an ITO coated coverslip with 80 nm Au beads that are used for image registration and alignment.

The basic procedure remains similar to our previous method (**Figure** **1A**). Cells expressing a fluorescent fusion protein were fixed with aldehydes, infiltrated with sucrose, and then frozen in liquid nitrogen. Cryosections of ∼100 nm thickness were cut with a diamond knife, picked up with a methylcellulose:sucrose solution and placed on coverslips coated with bare gold beads (80 nm) embedded in a thin layer of indium tin oxide (ITO). The ITO layer is thin enough so that it does not introduce noticeable optical loss, but provides enough electrical conductivity to avoid charging during subsequent SEM imaging. Specimens were then imaged using a standard 2D PALM microscope (**Figure** **1B**). After PALM imaging, the sections were stained with a 2% osmium tetroxide solution reduced with potassium ferrocyanide, then a 0.6% uranyl acetate solution followed by a 0.0075% lead solution in PVA. Sections were then spin-dried. The gold particles are both fluorescent and electron dense and thus were used to align and register the PALM and SEM datasets [9]. One problem with cryosections appears to be the significant folding and unevenness of the sections. Some folding may be reduced during the cryosection retrieval process [18], but some degree of folding is inevitable. In addition to obscuring structures of interest, folding produces regions of charging in the SEM that are not mitigated by ITO. We found that sputtering 1–2 nm of gold/palladium on top of the sample reduced charging on folds without noticeable loss of image quality or resolution.

### Single-color 2D correlative PALM/SEM of mitochondrial nucleoids, nuclear lamina, and peroxisomes

To compare our new staining procedure directly with our previous results we imaged cells expressing the mitochondrial transcription factor A (TFAM) fused to the photoactivatable fluorescent protein mEos2. TFAM is a marker for mitochondrial DNA-containing structures called nucleoids because in addition to its role as a transcription factor in mitochondrial RNA transcription, it acts in a histone-like manner to fold and compact the mitochondrial DNA [19], [20]. Mouse 3T3 fibroblast cells stably expressing TFAM-mEos2 were fixed, stained, and imaged as outlined in **Figure** **1A**.

**Figure 2:**
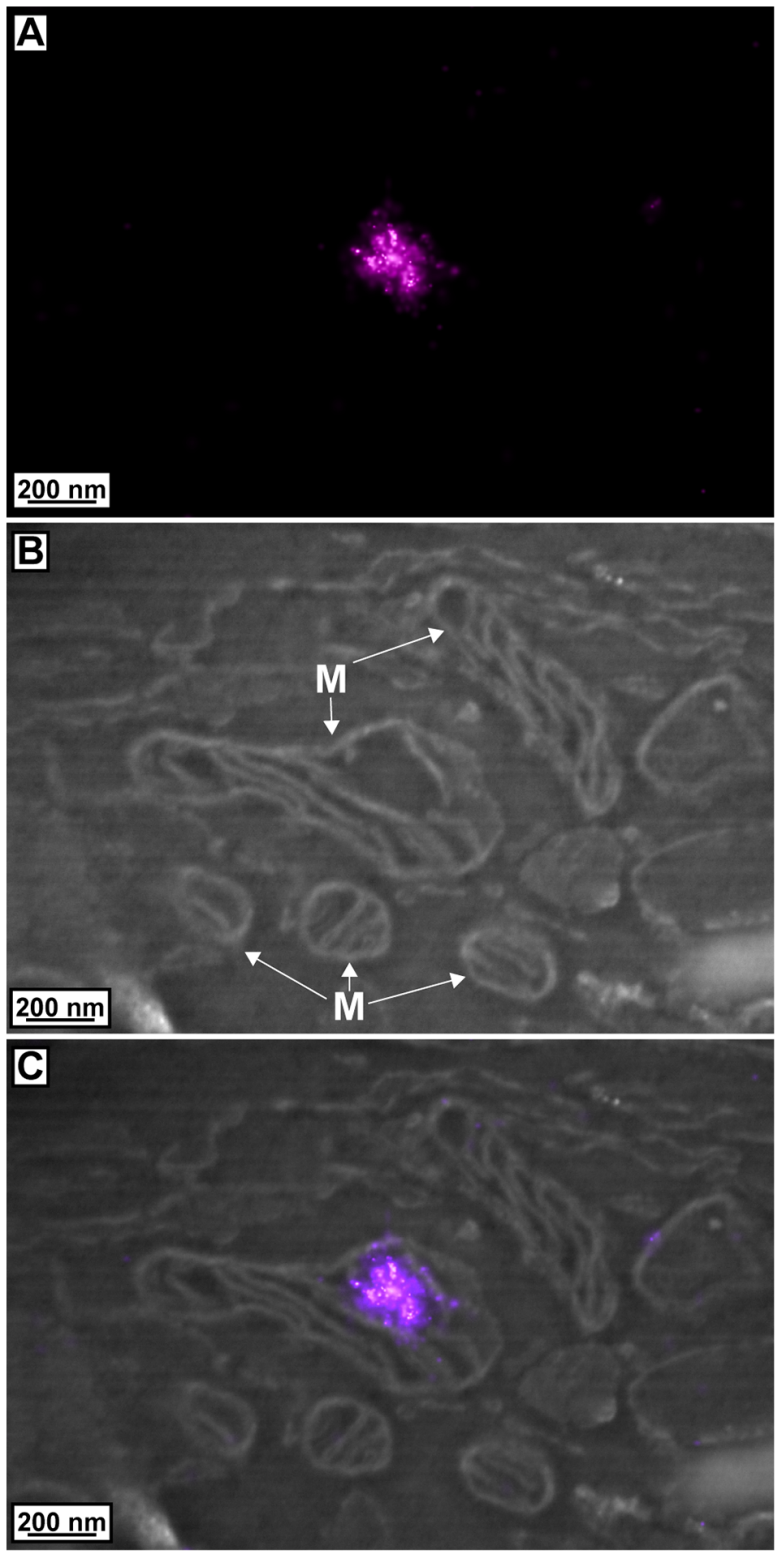
Correlated images of TFAM-mEos2 PALM data with electron micrographs. (**A**) PALM image of TFAM-mEos2. (**B**) SEM image of mitochondria (M) in the same area imaged in **A**. (**C**) Correlated image of TFAM-mEos2 PALM data with SEM data. TFAM-mEos2 resides in the mitochondrial matrix surrounded by boundary and cristae membranes.

**Figure 2** and **Figure** **S1** show the results of 2D correlative PALM/SEM imaging of cells expressing TFAM-mEos2. **Figure** **2A** and **Figure** **S1A** show the PALM image of TFAM-mEos2 while **Figure** **2B** and **Figure** **S1B** show the SEM image of the same area imaged by PALM. The quality of the SEM is very similar to, if not better than, our 3D method, and the quality of PALM also confirms good labeling density. The mitochondrial membranes, including the boundary membranes and cristae membranes, are well defined. There are several instances where the ∼10 nm space between cristae membranes is visible, which is close to the resolution limit of the SEM we used. **Figure** **2C** and **Figure** **S1C** show the correlated PALM/SEM image where, as observed previously, the mitochondrial nucleoid resides in a space within the mitochondrial matrix surrounded by cristae and the boundary membranes. The nucleoid is also the approximate size (100–200 nm) that was observed previously for nucleoids using PALM [9], [11] and other super-resolution methods [20].

Once we verified that our staining and contrasting procedure gave results comparable to or better than our original 3D method, we explored the versatility of this procedure to image other cellular structures. One cellular structure that has been imaged by several modes of super-resolution fluorescence microscopy and electron microscopy is the nuclear lamina [12], [21], [22]. The nuclear lamina is composed of proteins called lamins that form a fibrous meshwork inside the nuclear envelope that provides support to the nucleus [23]. Lamins interact with both integral membrane proteins of the inner nuclear envelope and chromatin, and defects in the nuclear lamina are linked to many human diseases [23]. To image the nuclear lamina we used a previously constructed fusion protein that has mEos2 genetically fused to the N-terminus of the lamin B1 protein [24]. This fusion construct was transiently transfected into 3T3 cells and processed for imaging as in **Figure** **1A**.

**Figure 3:**
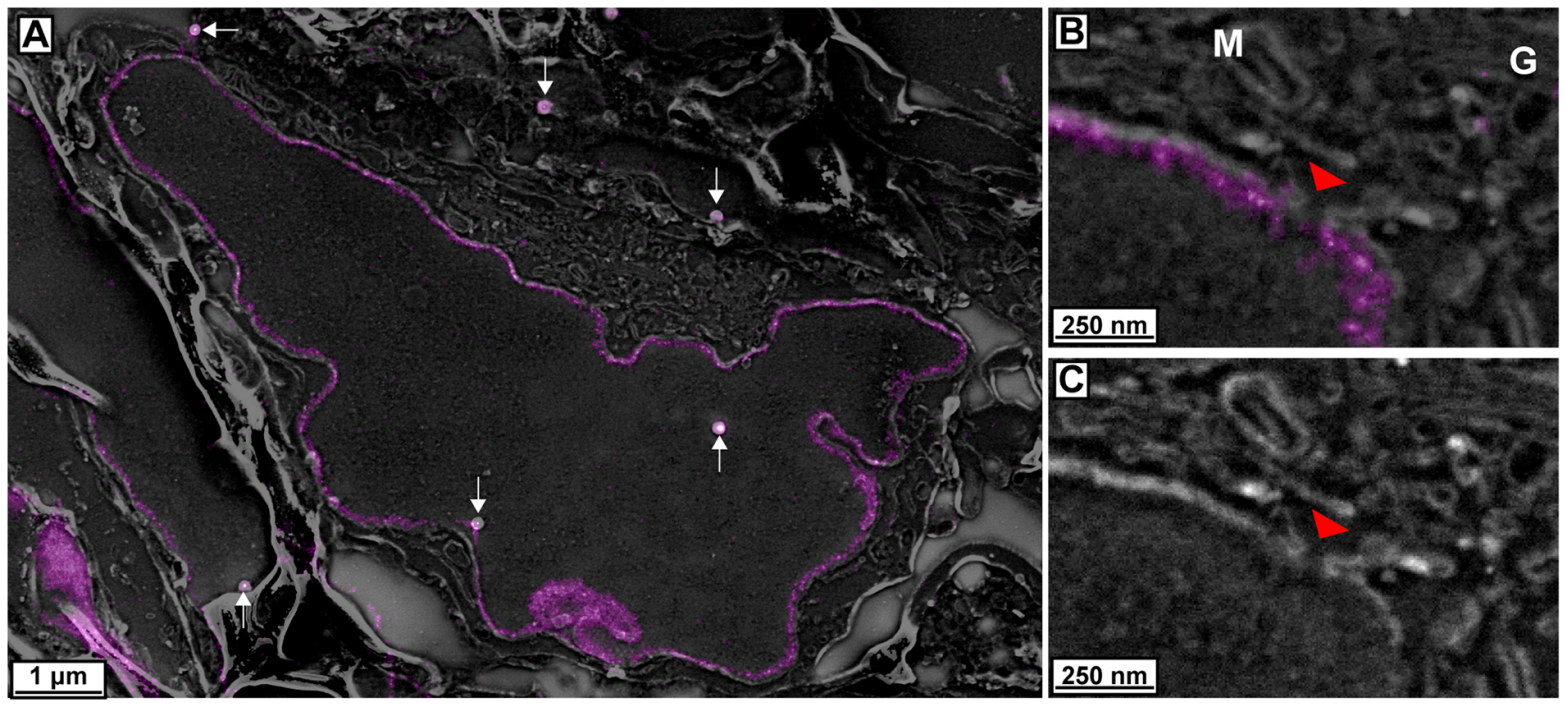
Correlated images of lamin B1-mEos2 PALM data with electron micrographs. (**A**) Correlated image of Lamin B1-mEos2 signal with electron micrograph. White arrows point to Au beads used for alignment. (**B**) Higher magnification view of an area from **A** showing a possible nuclear pore (red arrowhead) with corresponding gap in Lamin B1-mEos2 signal. (**C**) SEM only image of panel **B** to show cellular ultrastructure. M, mitochondrion. G, Golgi apparatus.

**Figure** **3** and **Figure** **S2** show the result of correlating lamin B1-mEos2 signal with SEM data. The magenta lamin B1-mEos2 signal from PALM follows the nucleoplasmic face of the nuclear envelope as seen in the SEM data, validating the resolution and localization precision of the PALM data. The relative continuity of the lamin signal indicates that the majority of the fluorescent proteins are preserved during processing. Also, the average thickness of the lamin signal is ∼50 nm, which is consistent with what is observed by other methods [23]. Membrane structure is well preserved throughout the cell with membranous organelles easily observable and identifiable including mitochondria, Golgi, and endoplasmic reticulum membranes. In multiple instances, there appears to be a gap in the lamin B1-mEos2 signal that corresponds to a gap in the nuclear envelope (**Figure** **3B, C** **red arrowheads**). This gap is likely the site of a nuclear pore complex (NPC) since it is the approximate width (120 nm) of a NPC [25] and a potential gap in the nuclear lamina at the site of NPCs has been suggested previously from other high-resolution fluorescence microscopy [21]. This particular example is an excellent demonstration of the power of this technique. The stochastic and spotty nature of immunolabeling EM would make it difficult to determine whether such a gap in signal is biologically real or the result of incomplete labeling, and diffraction-limited fluorescence microscopy would be unable to resolve whether a gap of less than 200 nm existed. Therefore, PALM provides the specificity and precise localization needed to examine the fine structural details of cellular morphology, and is made even more powerful by the additional context of electron microscopy.

Another structure we imaged was the peroxisome. One advantage of correlative microscopy is its ability to help identify or distinguish between morphologically similar cellular structures such as lysosomes and peroxisomes or other unique or rare structures [4], [5], [26]. Peroxisomes are 250–500 nm diameter spherical, membrane-enclosed organelles that contain at least 50 different enzymes involved in a variety of important biochemical processes in animal, plant, and yeast cells [27]. Proteins are targeted to peroxisomes by two main pathways that use either a carboxy terminal Serine-Lysine-Leucine (S-K-L) amino acid sequence or an amino terminal nine amino acid sequence [27]. To target mEos2 to the peroxisome we fused an S-K-L sequence to its carboxy terminus.

**Figure 4:**
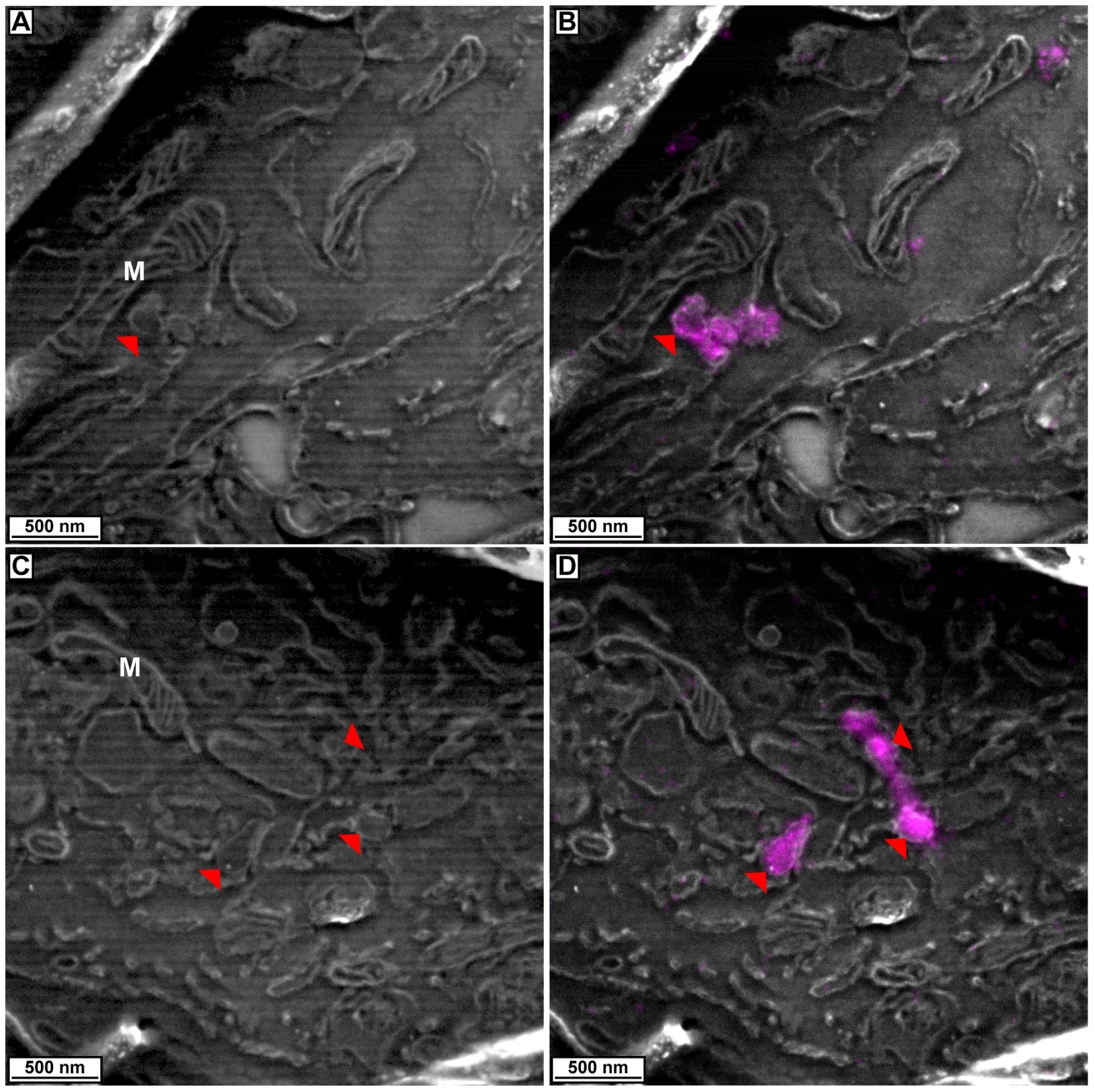
Correlated images of peroxisome-localized mEos2-SKL PALM data with electron micrographs. (**A**, **C**) SEM image of cells showing mitochondria (M) and other membranous organelles. Red arrowheads indicate peroxisomes as seen by overlaying PALM data of mEos2-SKL on electron micrographs (**B**, **D**).

Expression of mEos2-SKL in 3T3 cells was robust with a punctate pattern throughout the cytoplasm similar to what has been observed with other fluorescent protein-SKL fusion proteins (data not shown). The correlative PALM/SEM of peroxisomal localized mEos2 revealed the ability of this technique to help identify cellular structures (**Figure** **4** and **Figure** **S3**). SEM images of cells reveal well-defined morphological structures such as mitochondria (**Figure** **4A, C** and **Figure** **S3B**), however, throughout the cytoplasm are a variety of membranous organelles that may be any one of several morphologically similar structures. Overlaying the PALM data of the mEos2-SKL signal reveals those structures that are peroxisomes, which can be seen to vary in size in shape **(** **Figure** **4B, D** and **Figure** **S3C**). Thus, correlative PALM/SEM may provide a way to more accurately identify and distinguish structures within cells.

### Multi-color correlative PALM/SEM

Correlative PALM/SEM allows one to localize a fluorescent fusion protein within the context of an electron micrograph. However, there may be times when one wants to simultaneously localize multiple proteins. There are several examples of two-color PALM imaging in the literature [10] and one example involves examining the location of transcription factors relative to the nuclear periphery using lamin fluorescent protein fusions as markers [12]. Since two-color PALM imaging of the nuclear lamina was established, we performed two-color correlative microscopy with lamin A fused to the photoswitchable cyan fluorescent protein 2 (PS-CFP2) [28] and lamin B1-mEos2.

**Figure 5:**
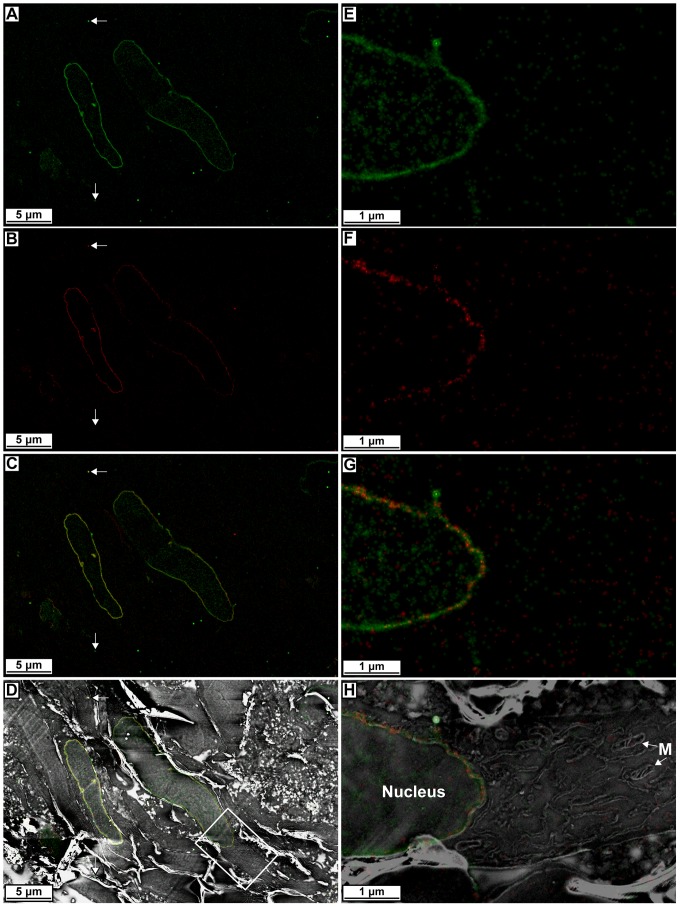
Two-color correlative PALM and SEM data of nuclear lamina proteins. (**A**) PALM image of lamin A-PS-CFP2. (**B**) PALM image of lamin B1-mEos2. (**C**) Combined lamin A-PS-CFP2 and lamin B1-mEos2 PALM data. (**D**) Combined two-color lamin protein PALM data correlated with electron micrograph. (**E-H**) Higher magnification views from boxed area in **D** of PALM image of lamin A-PS-CFP2 (**E**), PALM image of lamin B1-mEos2 (**F**), combined lamin A-PS-CFP2 and lamin B1-mEos2 PALM data (**G**), and combined two-color lamin protein PALM data correlated with electron micrograph (**H**). Note the cytoplasmic organelles such as mitochondria (M). White arrows in panels **A**-**D** indicate Au beads that are used to align the datasets.

We expressed both lamin B1-mEos2 and lamin A-PS-CFP2 in 3T3 cells by transient transfection and performed our correlative microscopy procedure. PALM imaging of lamin B1-mEos2 was performed first until the mEos2 signal was bleached, which was then followed by PALM imaging of lamin A-PS-CFP2. The PALM images of lamin A-PS-CFP2 and lamin B1-mEos2 both showed dense nuclear labeling and good co-localization to each other and the interior of the nuclear envelope (**Figure** **5** and **Figure** **S4**). Ultrastructure of the nuclear envelope and other cellular structures appear well defined. Thus, we demonstrate that different proteins can be simultaneously imaged by PALM and correlated to electron micrographs, which expands the versatility of this technique.

### Caged dye labeling of actin

Multiple methods have been developed that use small-molecule dyes for PALM microscopy [13], [29], [30]. PALM methods that use small molecule dyes are sometimes referred to as stochastic optical reconstruction microscopy (STORM) [29] and a modified version of STORM called direct-STORM (dSTORM) that requires a reducing environment [30]. An alternate class of caged dyes was originally demonstrated for PALM imaging [6] and recently improved [13], [14]. These dyes allow PALM to be performed in standard atmosphere-saturated buffers. The fluorescence of caged dyes is suppressed by a blocking moiety and can be uncaged enzymatically or, as in the case with PALM microscopy, uncaged through exposure to short wavelength (405 nm) light [13], [31]–[34]. This allows the stochastic activation of a sparse subset of fluorescent molecules for localization by PALM.

To determine if we could use caged dyes for correlative microscopy, we tested the caged dye carbo-rhodamine110 conjugated to phalloidin [14]. Phalloidin is a small molecule toxin from *Amanita phalloides* that binds tightly to F-actin and has commonly been conjugated to fluorescent molecules for imaging actin filaments in cells [35], [36]. Phalloidin labeled actin is an ideal structure to test correlative microscopy since it has been well characterized by multiple imaging modalities including EM and PALM [10], [37]. Although some fluorescent phallotoxins are membrane permeant [36], [37], we found the use of a detergent was necessary as is standard in most fluorescent phallotoxin labeling protocols [38]. Such permeabilization is a concern for maintaining ultrastructural integrity for electron microscopy since detergents disrupt cellular structures, especially membranes. To limit the disruptive effects of permeabilization treatment on cellular ultrastructure we used the detergent saponin. Saponin has been used in previous studies where one needs to permeabilize a cell membrane while retaining ultrastructure, such as immunolabeling EM [39].

Therefore, our dye labeling correlative procedure varied slightly from our standard fluorescent protein procedure. Phalloidin is able to bind F-actin even when concentrations up to 1% glutaraldehyde are used for fixation [37]. To get maximum labeling and structure preservation we reduced the concentration of glutaraldehyde in the initial fixative from 2% to 0.5%. Cells were then permeabilized with saponin and incubated with the caged dye-phalloidin conjugate. After dye labeling, the cells were taken through our standard procedure for cryosectioning and imaging.

**Figure 6:**
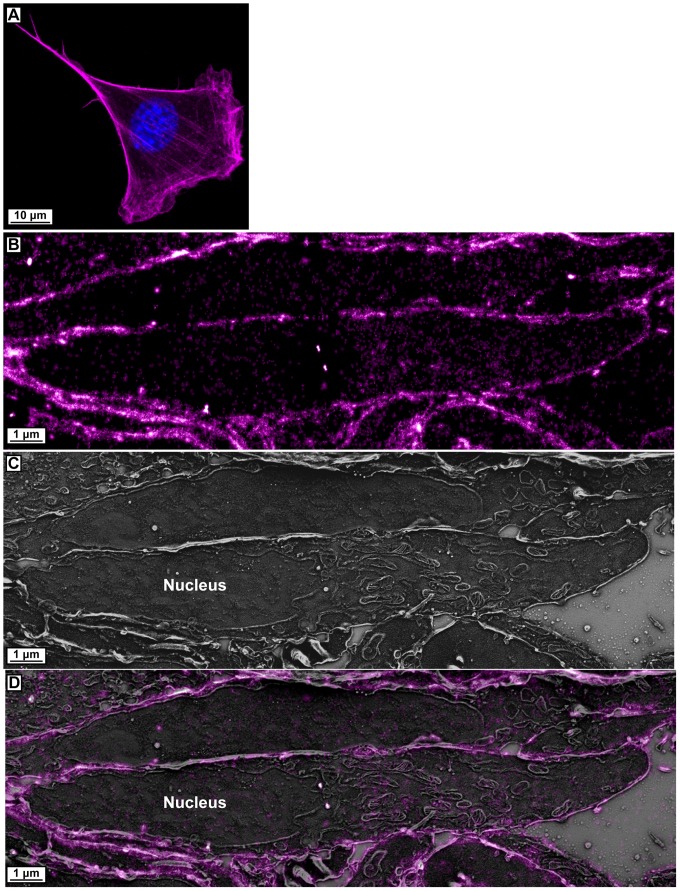
Caged dye-phalloidin labeling of actin and correlated PALM/SEM. (**A**) Maximum intensity projection image of confocal z-stack of a cell with actin labeled using NVOC_2_-carborhodamine110–PEG_8_–phalloidin (magenta) and the nuclear stain 4',6-diamidino-2-phenylindole (blue). (**B**) PALM image of caged dye labeled actin. (**C**) SEM of area imaged by PALM in panel **B**. (**D**) Correlated PALM and SEM of caged dye labeled actin.

**Figure 6A** shows whole cell imaging by confocal microscopy demonstrating the labeling of actin filaments by caged dye-phalloidin. **Figure** **6B–D** and **Figure** **S5** show the results of our correlative PALM/SEM imaging of caged dye labeled actin. The areas most densely labeled are just inside of the plasma membrane, which is consistent with results from other super-resolution fluorescence imaging studies [40]. Cytoplasmic filament structures are mostly absent due to the 100 nm thickness of the section, making it unlikely that a significant length of contiguous filament would be imaged.

## Discussion

In this work, we describe a correlative PALM and electron microscopy protocol that provides a high quality combination of fluorescence and EM images while using simpler instrumentation than our previous method. Additionally, we showed that this method can be used to image diverse proteins and cellular compartments. Another advance was the ability to perform multicolor correlative PALM/SEM, which is an advantage over other “single-color” correlative methods such as diaminobenzidine precipitation. We have not tested any photoactivatable fluorescent proteins other than mEos2 and PS-CFP2 for use in this protocol and it is possible that not every photoactivatable fluorescent protein will be functional using this method. However, a bigger limitation to multicolor PALM imaging is finding fluorophores that are well separated spectrally.

We also demonstrated that this method can be used for imaging dyes by labeling actin with a caged dye-phalloidin conjugate. Caged dyes present intriguing possibilities for new applications because they could be used to label a variety of objects. Such dyes can be conjugated to modified nucleotides or amino acids to examine processes such as DNA replication [13], [41], RNA transcription [42], and protein translation [43], [44] or to localize specific nucleotide sequences, such as in fluorescence *in situ* hybridization. Another potential application is to use caged dyes in conjunction with protein labeling technologies such as SNAP-tag [45] or HaloTag [46]. Caged dyes may also be designed to cover a wider fluorescence spectrum than fluorescent proteins, especially in the red spectrum [14].

Potential exists for this correlative PALM/SEM procedure to be combined with other labeling protocols. Xu et al. used cryosections to perform imaging with antibody attached photoactivatable dyes, and it remains to be explored how well this approach can preserve EM ultrastructure [47]. Also, since we use osmium tetroxide for staining, our correlative method could be combined with a diaminobenzidine precipitation method to add another layer of information for multicomponent structures. Performing serial section correlative PALM/SEM is possible, but the main barrier remains obtaining consistent ribbons of consecutive sections during cryosectioning. Serial section correlative PALM/SEM would provide a way to perform a high-resolution array tomography-like procedure for the examination of larger tissue volumes [48].

The original development of this procedure involved the use of transmission electron microscopy (TEM) instead of SEM. However, correlative PALM/TEM has several difficulties. If a section is placed on a TEM grid before PALM imaging, there is significant risk of breakage of the support film, obscuration of parts of the sample by the grid, and unevenness of the TEM grid that results in parts of the region of interest being out of PALM focus. Alternatively, the sections can be placed on a pioloform-coated coverslip and then transferred to a TEM grid by floating the pioloform off the coverslip; however, this is technically demanding. Thus, being able to have sections on a coverslip for both PALM and SEM imaging is a major advantage of this procedure, but correlating PALM images to TEM data is feasible [6] if an SEM is not available or slightly higher resolution EM is required.

In summary, this protocol provides a way to map multicomponent cellular structures with high localization precision, high specificity, and detailed ultrastructural context. Also, using a simpler imaging system will make this protocol more accessible to a wider range of researchers and the versatility of this technique will be valuable for answering multifaceted biological questions.

## Methods

### Plasmids

Plasmid Lamin B1-mEos2 was a gift from L. Looger and is described in [24]. Plasmid Lamin A-PS-CFP2 was a gift from J. Yao and is described in [12]. Plasmid mEos2-SKL was constructed using standard molecular cloning techniques [49] and was verified by nucleotide sequencing. The amino acid sequence SGSKL was inserted at the C-terminus of mEos2 using the primer 5′-GATGACGCGGCCGCTTACAGCTTGGATCCGGATCGTCTGGCATTGTCAGGCAATCCAGAATG-3′. The SKL containing primer that annealed to the C-terminal coding region of mEos2 and a flanking primer that annealed to the N-terminal coding region of mEos2 were used to amplify a fragment containing the mEos2-SGSKL sequence. The fragment was digested with restriction enzymes XhoI and NotI and ligated into common sites of similarly digested sites of the cloning vector mEos2-N1, which had the original mEos2 digested away and the backbone purified.

### Transfection and expression

Mouse fibroblast 3T3 Switch cells (Life Technologies, Carlsbad, CA) were maintained in Dulbecco's modified eagle's medium, 10% normal calf serum, 1 mM sodium pyruvate, 2 mM L-alanyl-L-glutamine, and 50 µg/ml hygromycin B. TFAM-mEos2 was stably maintained and expressed in 3T3 Switch cells as described [11]. All other plasmids were transfected into 3T3 Switch cells using a nucleofector system (Lonza, Switzerland) as described [50].

### Cell fixation and cryosectioning

Cells were fixed and prepared for Tokuyasu cryosectioning as described [9] except for cells labeled with the caged dye-phalloidin. Cryosectioning was performed as [9].

### PALM imaging

Coverslips were prepared in the following way. First, 80 nm bare Au nanospheres (part# 790120-010, Microspheres-Nanospheres, Cold Spring, NY) were deposited onto 25 mm #1.5 coverslips (Warner Instruments, Hamden, Connecticut). Then a thin layer of Indium Tin Oxide (ITO) was sputtered over the coverslip using a Denton Explorer sputtering system (Denton Vacuum, Moorestown, New Jersey). The exact thickness of the ITO layer was not measured, instead we measured the electrical resistance between two points across the coverslip, which was ∼2 kOhm. The optical loss from the ITO layer was less than 5%. Before PALM imaging, sections on coverslips were washed with PBS and treated with 0.5% sodium borohydride in 100 mM phosphate buffer pH 7.4.

Typical PALM data acquisition consisted of 40,000–80,000 frames using an iXon DU-897E EM-CCD camera (Andor, UK). Acquisition was performed in frame transfer mode and the laser excitation was constantly ON during acquisition. Power densities, filters, and the durations of activation and exposure pulses for different wavelength channels are listed in **Table** **1**.

**Table 1:** PALM imaging acquisition parameters.

	Activation	psCFP2 excitation	mEos2/Rh110 excitation
Wavelength (nm)	405	488	561
Power density (W/cm^2^)	∼2÷10	∼400	∼1000
Exposure Time (ms)	0.5÷50	50	50
Laser source (model and manufacturer)	CUBE 405–50 (Coherent, Santa Clara, CA)	Cyan 488 (Newport, Spectra Physics, Santa Clara, CA)	CL561-150-O (CrystaLaser, Reno, NV)
Filters (Semrock, Rochester, NY)		NF01-405/488/561/635 quad-notch filter	
		LP02-488RS, FF01-520/35	LP02-568RU, FF01-593/40

Power densities, lasers, filters, and the durations of activation and exposure pulses for different wavelength channels during PALM acquisition.

Two-channel PALM image registration was performed using Au nanoparticles. The same nanoparticles were observed under 561 nm illumination and 488 nm illumination, which allowed for registration of the two channels as described [11].

When a large set of fiducial Au nanoparticles was detectable across the field of view (n≥20), we registered the images using a POLYWARP 1 image transformation defined as:
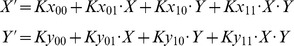



This bilinear interpolation transformation is an affine transformation plus an extra XY cross term and is available in most image processing packages. We used the POLYWARP function with degree 1 from the IDL image processing language (http://www.exelisvis.com/docs/POLYWARP.html). This procedure reduced the average registration error to ∼5 nm. These Au nanoparticles can also be easily identified in SEM micrographs, so we used the same procedure to register PALM images to SEM images.

### Staining and SEM

Sections were stained in a solution of 2% osmium tetroxide and 1.6% potassium ferrocyanide in 100 mM phosphate buffer pH 7.4 for 15 min. Sections were then washed three times for 2 min in water and then stained with 0.6% uranyl acetate in 1.6% polyvinyl alcohol (PVA) for 15 min. After three brief washes in 2% PVA, the sections were stained for 15 min in a solution of 0.0075% Sato's lead [51] solution in 2% PVA. Coverslips were dried using a spin coater (Laurell Technologies, North Wales, PA) operated at 5000 rpm for 10 sec. A thin coating of Au/Pd was added using Precision Etching and Coating System (Model 682 PECS; Gatan, Pleasanton, CA). SEM images were acquired using an accelerating voltage of 1.5 KeV using either a Merlin, Sigma, or Ultra system (Carl Zeiss, Germany). The working distance was typically 3–5 mm with 200–500 pA of beam current and the in-lens detector captured back scattered electrons for a period of ∼100 μsec/pixel.

### Phalloidin labeling

3T3 Switch cells were fixed with 4% paraformaldehyde and 0.5% glutaraldehyde in PBS pH 7.4 for 10 min. Residual aldehyde groups were inactivated with 0.1% sodium borohydride in 100 mM phosphate buffer pH 7.4 for 7 min. Cells were then permeabilized in a solution of 3% bovine serum albumin and 0.1% saponin in PBS for 15 min. Cells were labeled with 2 μM NVOC_2_-carborhodamine110–PEG_8_–phalloidin in PBS for 1 hr. After a brief wash with PBS the cells were scraped, pelleted, and processed for cryosectioning.

### Confocal imaging

3T3 Switch cells were grown on #1.5 borosilicate coverglass in Lab-Tek II chambers (Nunc, Rochester, NY) and stained with 2 μM NVOC_2_-carborhodamine110–PEG_8_–phalloidin according to our phalloidin labelling protocol (see Methods). After phalloidin labelling, nuclei were stained with 300 nM 4′,6-diamidino-2-phenylindole (DAPI) in PBS for 5 min, followed by a brief wash in PBS and immediate imaging. Imaging was performed using a Zeiss 510 META confocal microscope (Carl Zeiss, Germany) with a 100X 1.4-numerical aperture Plan-Apochromat objective. A 405-nm diode laser was used to excite DAPI and simultaneously uncage NVOC_2_-carborhodamine110. A 15-mW DPSS 561-nm diode laser was used to excite carborhodamine110. The image represents a maximum intensity projection created using ImageJ (NIH) from a 1,024-by-1,024, 12-bit *z*-stack acquisition with sequential scanning at 0.8 μm steps.

## Supporting Information

Figure S1 **Larger field of view correlated images of TFAM-mEos2 PALM data with electron micrographs.** (**A**) Lower magnification PALM image of TFAM-mEos2 with a larger field of view than the selected area shown in Figure 2A. (**B**) Lower magnification SEM image with a larger field of view than the selected area shown in Figure 2B. (**C**) Lower magnification registered and overlaid PALM and SEM images with a larger field of view than the selected area shown in Figure 2C.
(ZIP)

Figure S2 **Larger field of view correlated images of lamin B1-mEos2 PALM data with electron micrographs.** (**A**) Lower magnification PALM image of lamin B1-mEos2 with a larger field of view than the selected area shown in Figure 3. (**B**) Lower magnification SEM image with a larger field of view than the selected area shown in Figure 3. (**C**) Lower magnification registered and overlaid PALM and SEM images with a larger field of view than the selected area shown in Figure 3.
(ZIP)

Figure S3 **Larger field of view correlated images of peroxisome-localized mEos2-SKL PALM data with electron micrographs.** (**A**) Lower magnification PALM image of peroxisome localized mEos2-SKL with a larger field of view than the selected areas shown in Figure 4. (**B**) Lower magnification SEM image with a larger field of view than the selected areas shown in Figure 4. (**C**) Lower magnification registered and overlaid PALM and SEM images with a larger field of view than the selected areas shown in Figure 4.
(ZIP)

Figure S4 **Larger field of view two-color correlative PALM and SEM data of nuclear lamina proteins.** (**A**) Lower magnification PALM image of lamin A-PS-CFP2 with a larger field of view than the selected areas shown in Figure 5. (**B**) Lower magnification PALM image of lamin B1-mEos2 with a larger field of view than the selected areas shown in Figure 5. (**C**) Lower magnification of combined lamin A-PS-CFP2 and lamin B1-mEos2 PALM image with a larger field of view than the selected areas shown in Figure 5. (**D**) Lower magnification SEM image with a larger field of view than the selected areas shown in Figure 5. (**E**) Lower magnification two-color lamin protein PALM image registered and overlaid with the corresponding SEM image with a larger field of view than the selected areas shown in Figure 5.
(ZIP)

Figure S5 **Larger field of view correlated images of caged dye-phalloidin labeled actin PALM data with electron micrographs.** (**A**) Lower magnification PALM image of caged dye labeled actin with a larger field of view than the selected area shown in Figure 6B. (**B**) Lower magnification SEM image with a larger field of view than the selected area shown in Figure 6C. (**C**) Lower magnification registered and overlaid PALM and SEM images with a larger field of view than the selected area shown in Figure 6D.
(ZIP)
