# Supplementary figures and images for: Correlative Photoactivated Localization and Scanning Electron Microscopy
Source: PLoS One. 2013 Oct 25;8(10):e77209. doi: 10.1371/journal.pone.0077209 (PMC3808397; doi:10.1371/journal.pone.0077209)

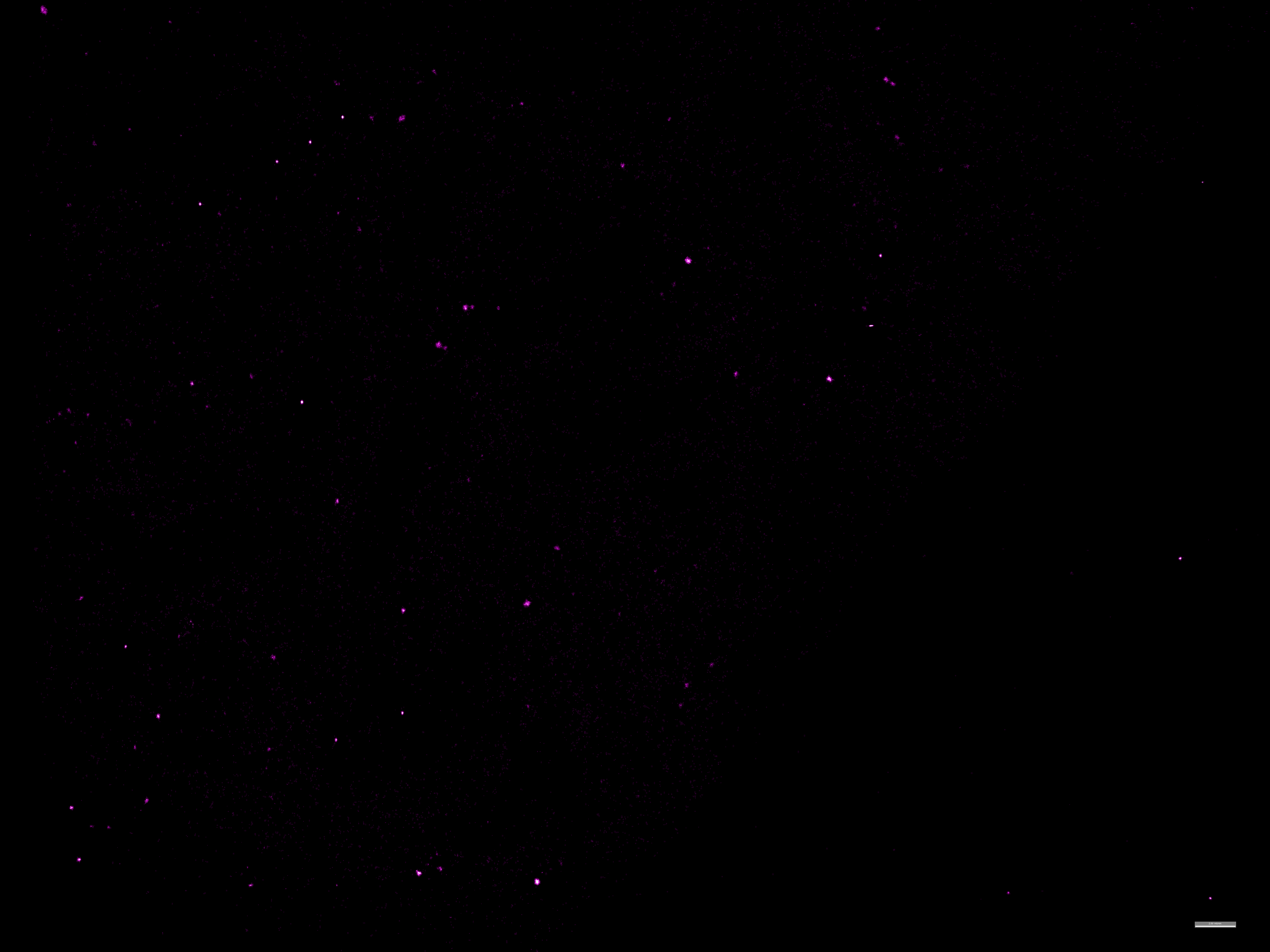

Supplement: Figure S1 — Larger field of view correlated images of TFAM-mEos2 PALM data with electron micrographs. (A) Lower magnification PALM image of TFAM-mEos2 with a larger field of view than the selected area shown in Figure 2A. (B) Lower magnification SEM image with a larger field of view than the selected area shown in Figure 2B. (C) Lower magnification registered and overlaid PALM and SEM images with a larger field of view than the selected area shown in Figure 2C. (ZIP) [file pone.0077209.s001.zip › Figure-S1/Figure S1A.tif]

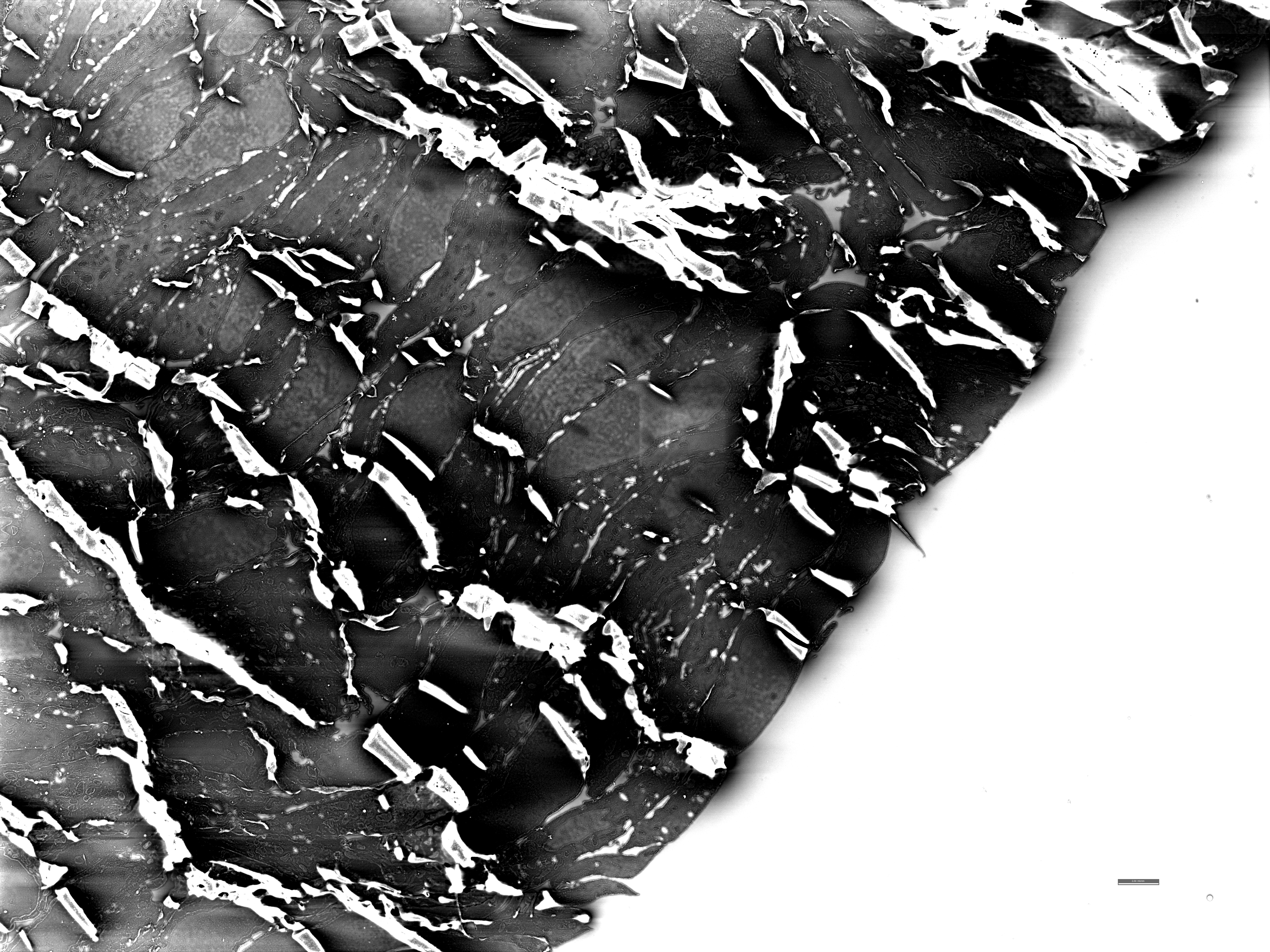

Supplement: Figure S1 — Larger field of view correlated images of TFAM-mEos2 PALM data with electron micrographs. (A) Lower magnification PALM image of TFAM-mEos2 with a larger field of view than the selected area shown in Figure 2A. (B) Lower magnification SEM image with a larger field of view than the selected area shown in Figure 2B. (C) Lower magnification registered and overlaid PALM and SEM images with a larger field of view than the selected area shown in Figure 2C. (ZIP) [file pone.0077209.s001.zip › Figure-S1/Figure S1B.tif]

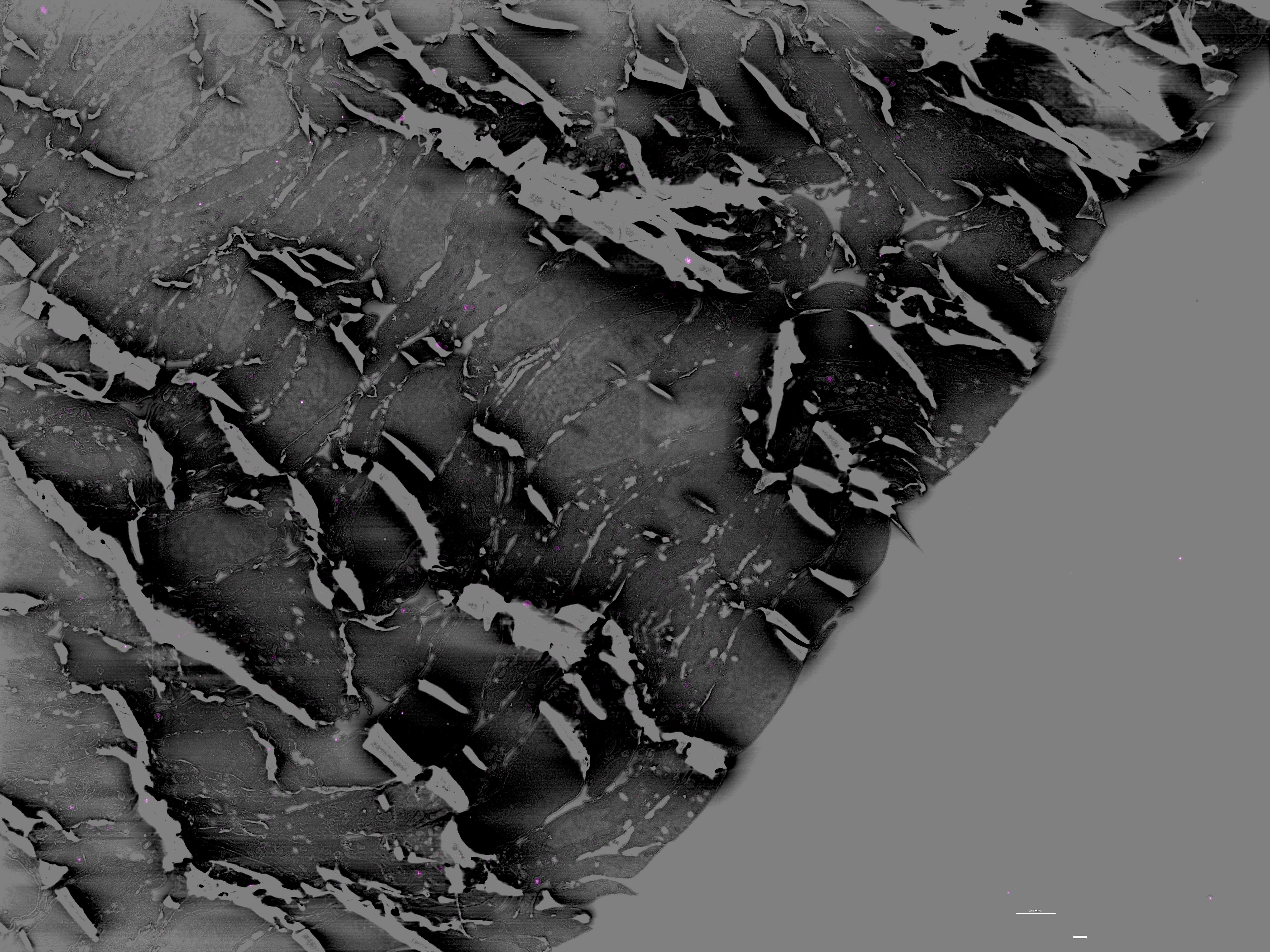

Supplement: Figure S1 — Larger field of view correlated images of TFAM-mEos2 PALM data with electron micrographs. (A) Lower magnification PALM image of TFAM-mEos2 with a larger field of view than the selected area shown in Figure 2A. (B) Lower magnification SEM image with a larger field of view than the selected area shown in Figure 2B. (C) Lower magnification registered and overlaid PALM and SEM images with a larger field of view than the selected area shown in Figure 2C. (ZIP) [file pone.0077209.s001.zip › Figure-S1/Figure S1C.tif]

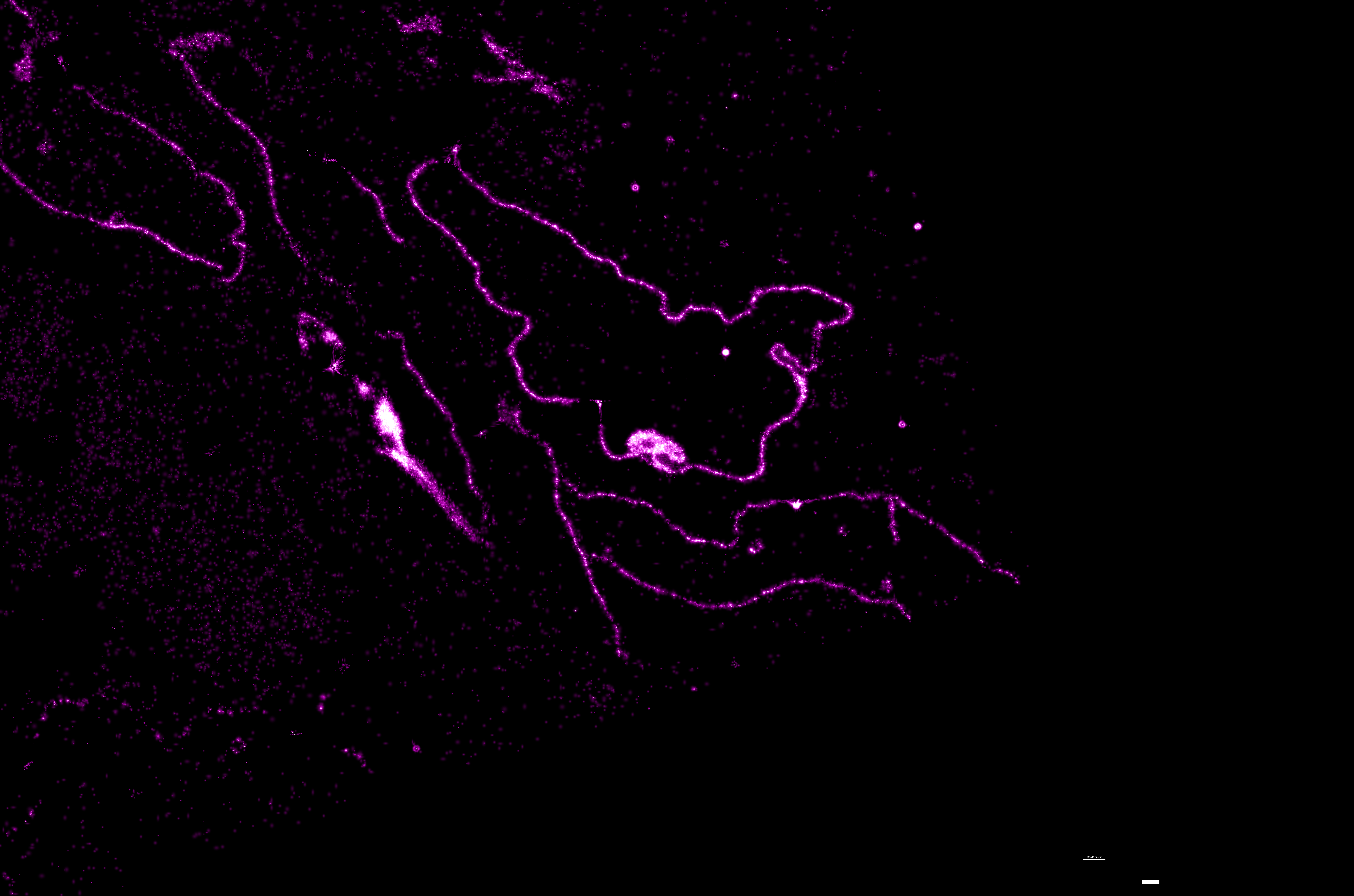

Supplement: Figure S2 — Larger field of view correlated images of lamin B1-mEos2 PALM data with electron micrographs. (A) Lower magnification PALM image of lamin B1-mEos2 with a larger field of view than the selected area shown in Figure 3. (B) Lower magnification SEM image with a larger field of view than the selected area shown in Figure 3. (C) Lower magnification registered and overlaid PALM and SEM images with a larger field of view than the selected area shown in Figure 3. (ZIP) [file pone.0077209.s002.zip › Figure-S2/Figure S2A.tif]

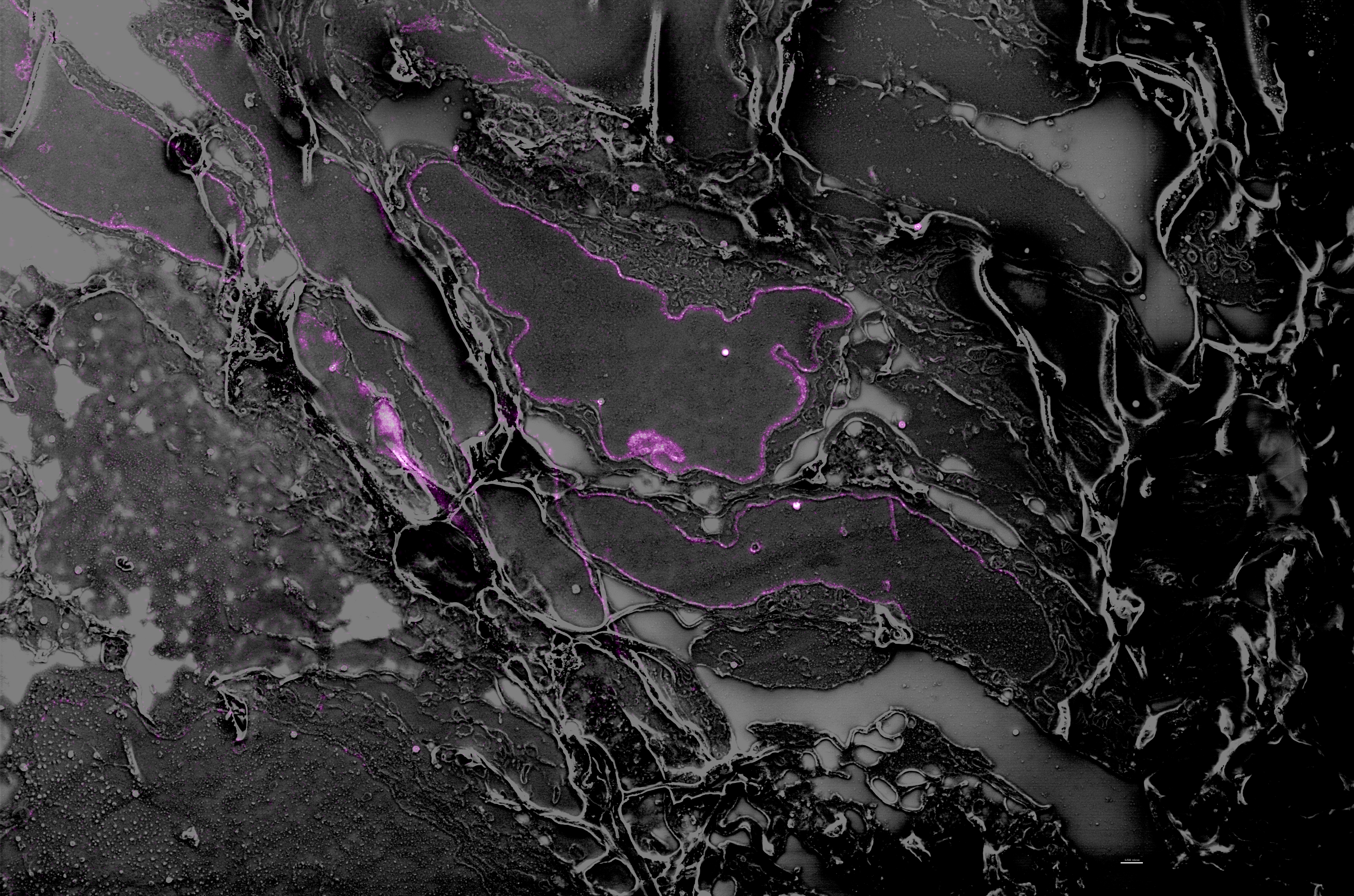

Supplement: Figure S2 — Larger field of view correlated images of lamin B1-mEos2 PALM data with electron micrographs. (A) Lower magnification PALM image of lamin B1-mEos2 with a larger field of view than the selected area shown in Figure 3. (B) Lower magnification SEM image with a larger field of view than the selected area shown in Figure 3. (C) Lower magnification registered and overlaid PALM and SEM images with a larger field of view than the selected area shown in Figure 3. (ZIP) [file pone.0077209.s002.zip › Figure-S2/Figure S2C.tif]

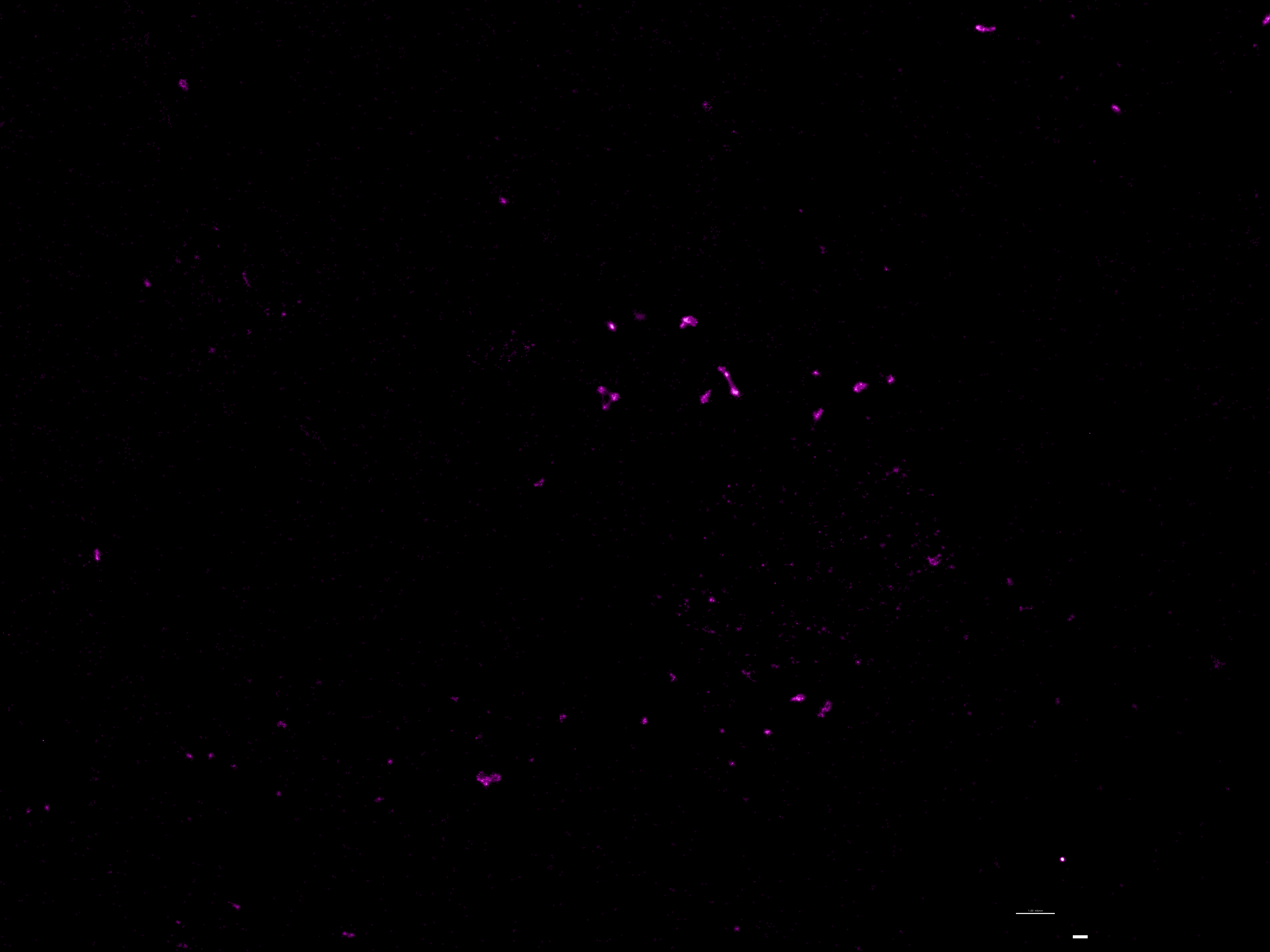

Supplement: Figure S3 — Larger field of view correlated images of peroxisome-localized mEos2-SKL PALM data with electron micrographs. (A) Lower magnification PALM image of peroxisome localized mEos2-SKL with a larger field of view than the selected areas shown in Figure 4. (B) Lower magnification SEM image with a larger field of view than the selected areas shown in Figure 4. (C) Lower magnification registered and overlaid PALM and SEM images with a larger field of view than the selected areas shown in Figure 4. (ZIP) [file pone.0077209.s003.zip › Figure-S3/Figure S3A.tif]

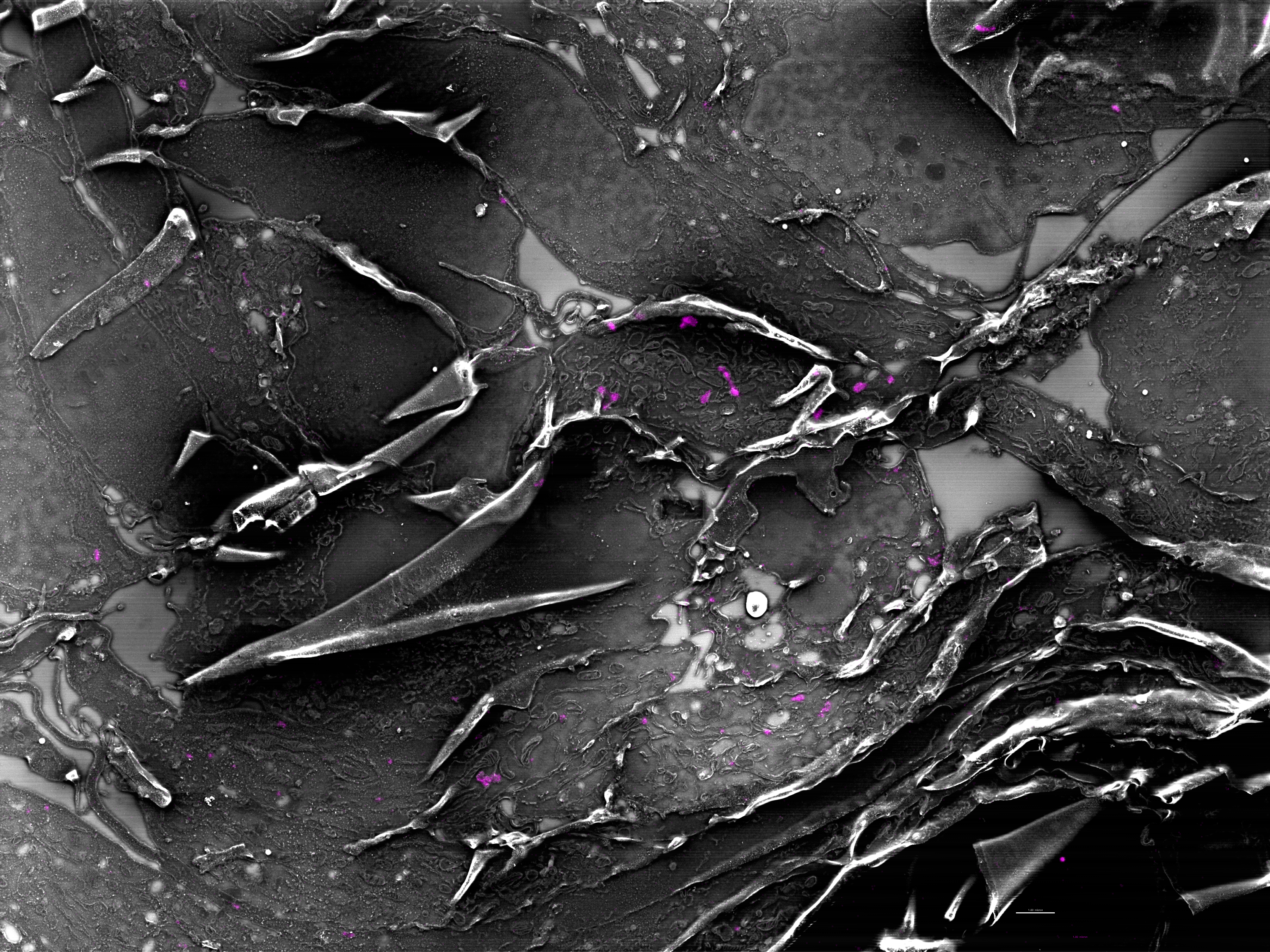

Supplement: Figure S3 — Larger field of view correlated images of peroxisome-localized mEos2-SKL PALM data with electron micrographs. (A) Lower magnification PALM image of peroxisome localized mEos2-SKL with a larger field of view than the selected areas shown in Figure 4. (B) Lower magnification SEM image with a larger field of view than the selected areas shown in Figure 4. (C) Lower magnification registered and overlaid PALM and SEM images with a larger field of view than the selected areas shown in Figure 4. (ZIP) [file pone.0077209.s003.zip › Figure-S3/Figure S3C.tif]

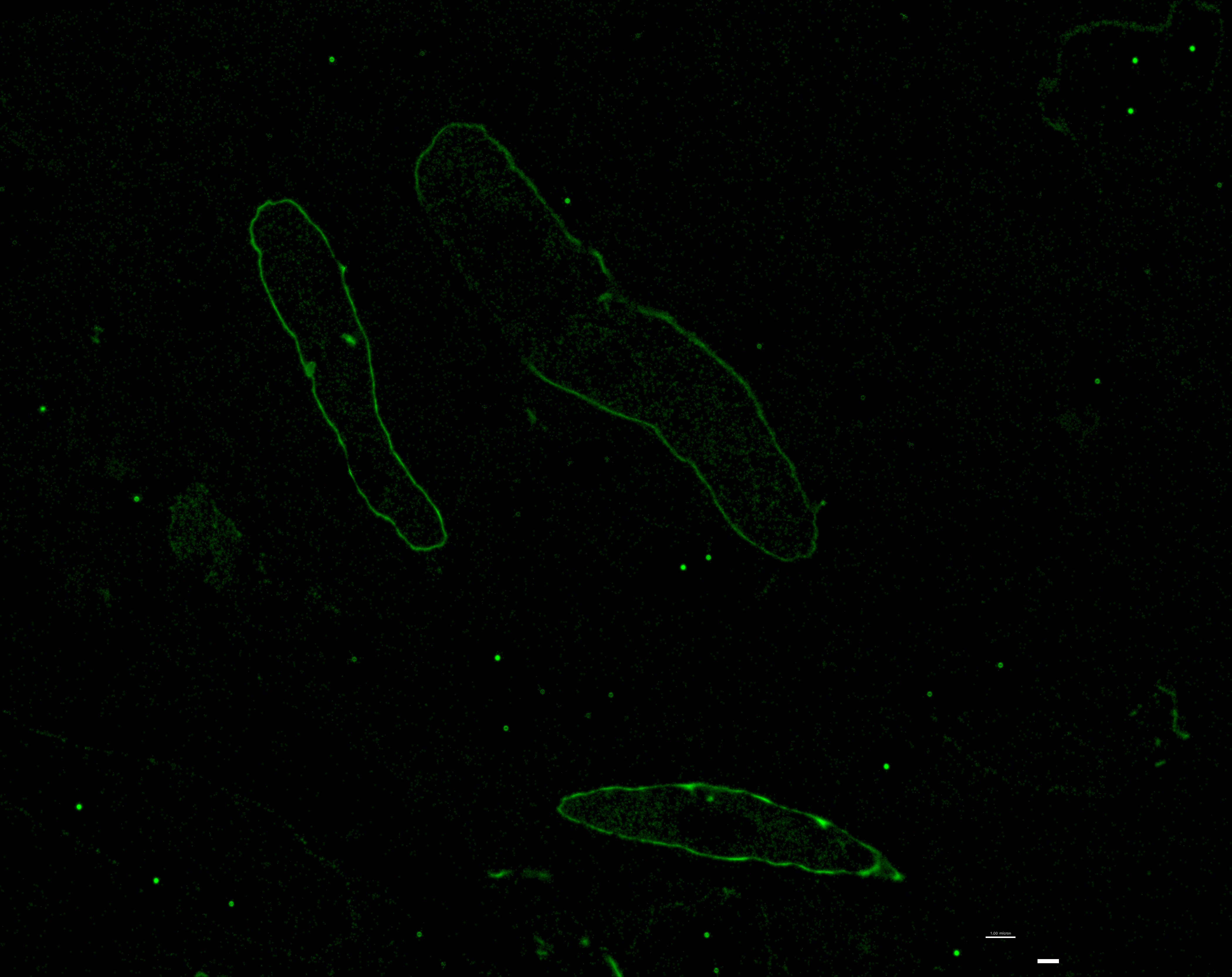

Supplement: Figure S4 — Larger field of view two-color correlative PALM and SEM data of nuclear lamina proteins. (A) Lower magnification PALM image of lamin A-PS-CFP2 with a larger field of view than the selected areas shown in Figure 5. (B) Lower magnification PALM image of lamin B1-mEos2 with a larger field of view than the selected areas shown in Figure 5. (C) Lower magnification of combined lamin A-PS-CFP2 and lamin B1-mEos2 PALM image with a larger field of view than the selected areas shown in Figure 5. (D) Lower magnification SEM image with a larger field of view than the selected areas shown in Figure 5. (E) Lower magnification two-color lamin protein PALM image registered and overlaid with the corresponding SEM image with a larger field of view than the selected areas shown in Figure 5. (ZIP) [file pone.0077209.s004.zip › Figure-S4/Figure S4A.tif]

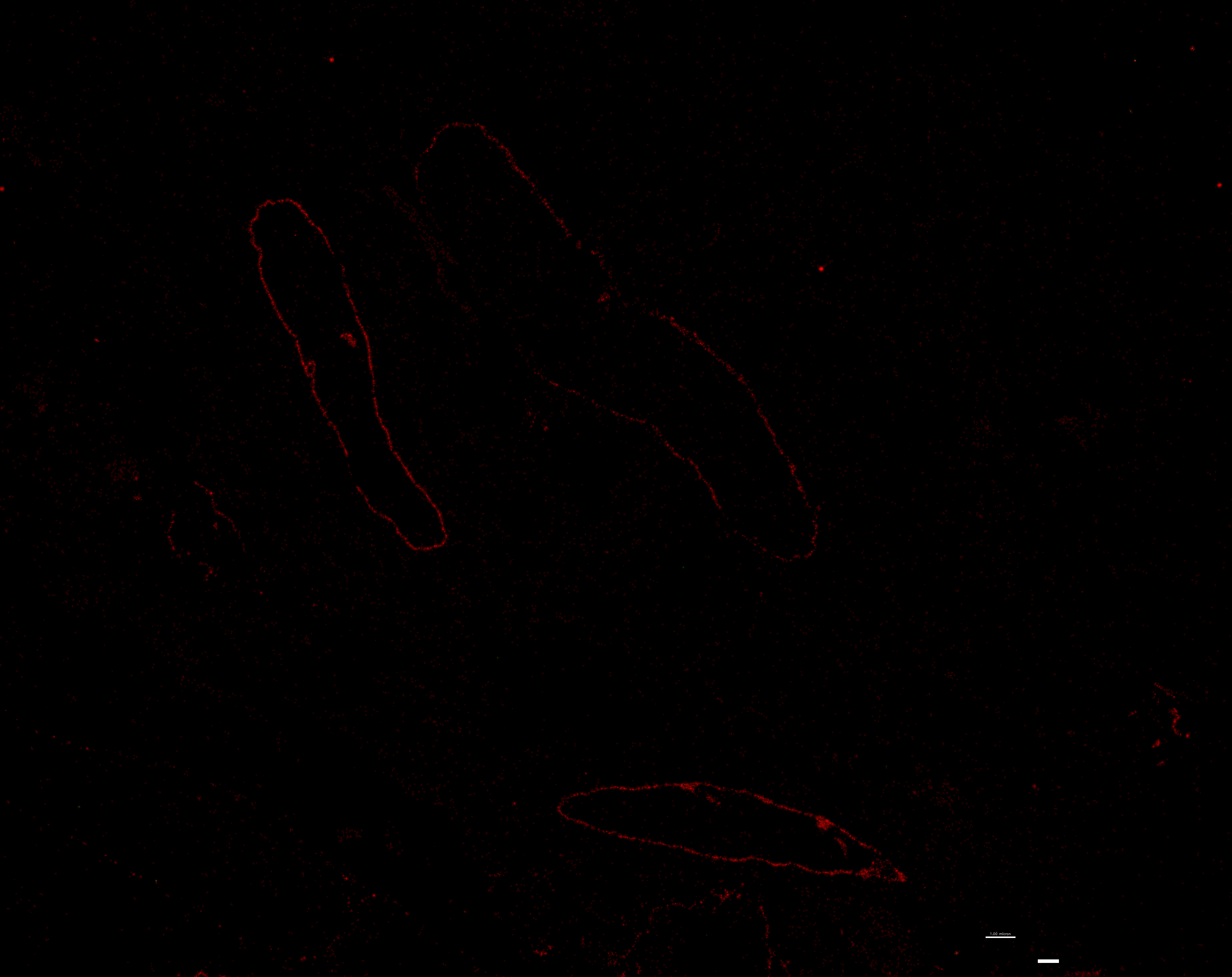

Supplement: Figure S4 — Larger field of view two-color correlative PALM and SEM data of nuclear lamina proteins. (A) Lower magnification PALM image of lamin A-PS-CFP2 with a larger field of view than the selected areas shown in Figure 5. (B) Lower magnification PALM image of lamin B1-mEos2 with a larger field of view than the selected areas shown in Figure 5. (C) Lower magnification of combined lamin A-PS-CFP2 and lamin B1-mEos2 PALM image with a larger field of view than the selected areas shown in Figure 5. (D) Lower magnification SEM image with a larger field of view than the selected areas shown in Figure 5. (E) Lower magnification two-color lamin protein PALM image registered and overlaid with the corresponding SEM image with a larger field of view than the selected areas shown in Figure 5. (ZIP) [file pone.0077209.s004.zip › Figure-S4/Figure S4B.tif]

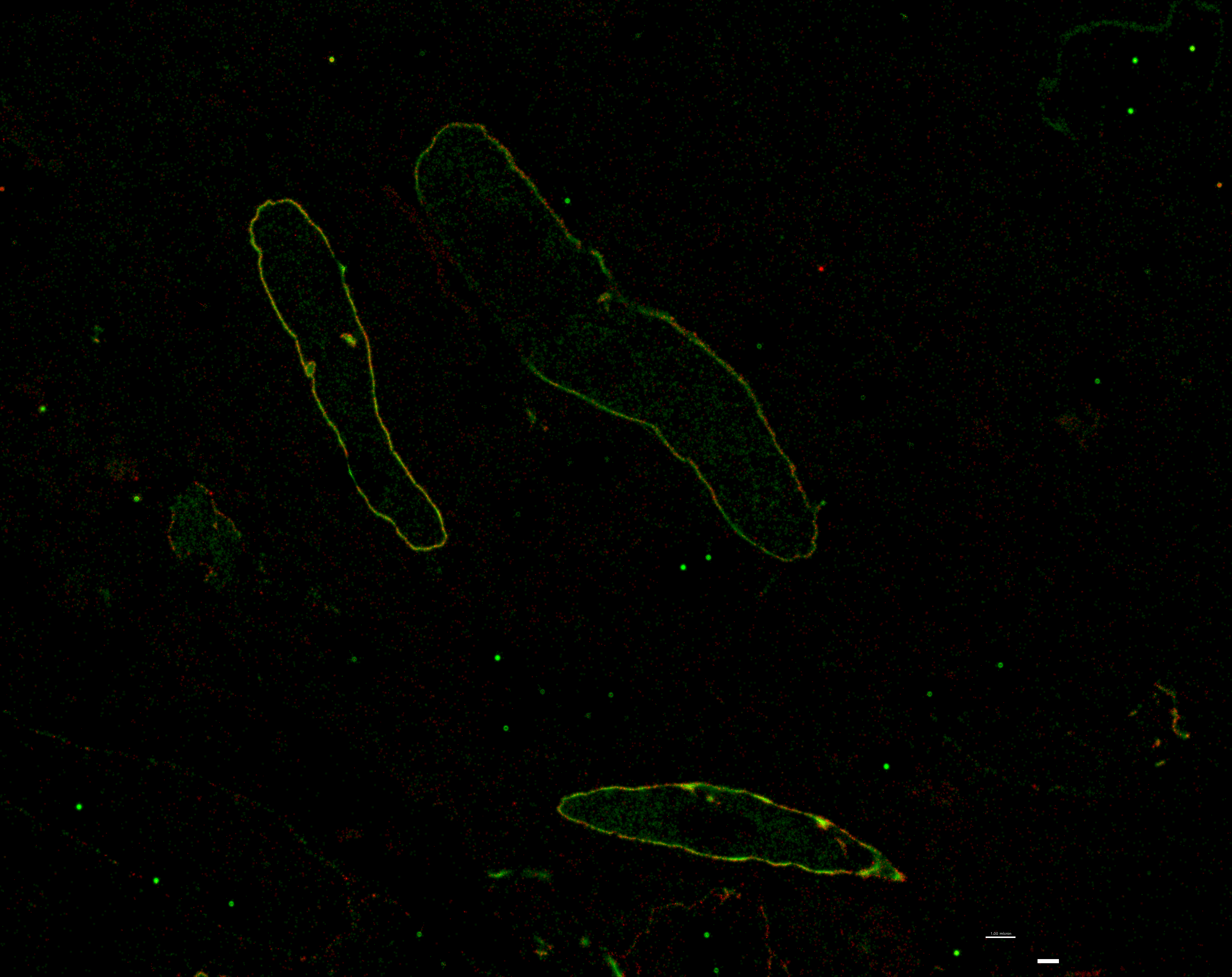

Supplement: Figure S4 — Larger field of view two-color correlative PALM and SEM data of nuclear lamina proteins. (A) Lower magnification PALM image of lamin A-PS-CFP2 with a larger field of view than the selected areas shown in Figure 5. (B) Lower magnification PALM image of lamin B1-mEos2 with a larger field of view than the selected areas shown in Figure 5. (C) Lower magnification of combined lamin A-PS-CFP2 and lamin B1-mEos2 PALM image with a larger field of view than the selected areas shown in Figure 5. (D) Lower magnification SEM image with a larger field of view than the selected areas shown in Figure 5. (E) Lower magnification two-color lamin protein PALM image registered and overlaid with the corresponding SEM image with a larger field of view than the selected areas shown in Figure 5. (ZIP) [file pone.0077209.s004.zip › Figure-S4/Figure S4C.tif]

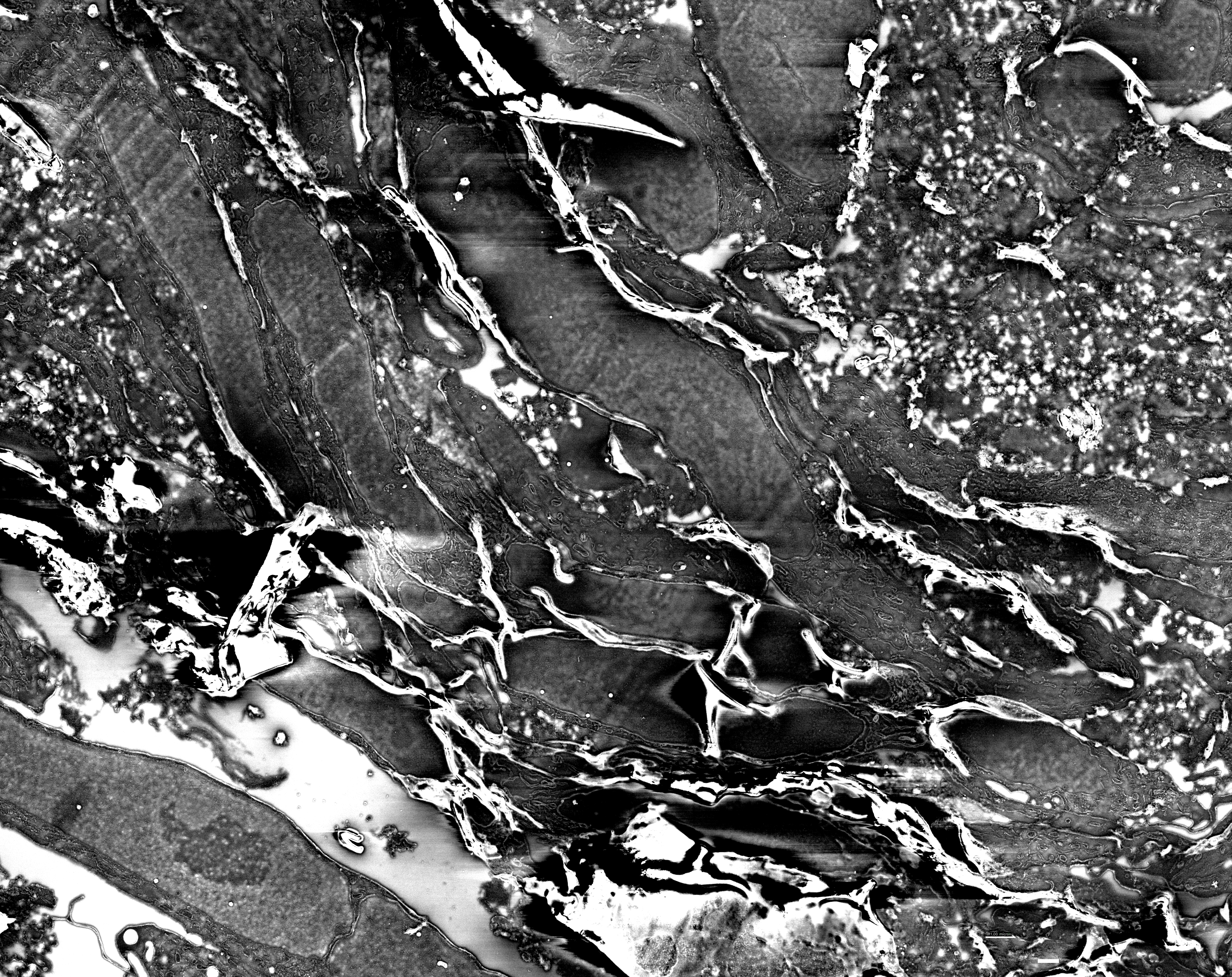

Supplement: Figure S4 — Larger field of view two-color correlative PALM and SEM data of nuclear lamina proteins. (A) Lower magnification PALM image of lamin A-PS-CFP2 with a larger field of view than the selected areas shown in Figure 5. (B) Lower magnification PALM image of lamin B1-mEos2 with a larger field of view than the selected areas shown in Figure 5. (C) Lower magnification of combined lamin A-PS-CFP2 and lamin B1-mEos2 PALM image with a larger field of view than the selected areas shown in Figure 5. (D) Lower magnification SEM image with a larger field of view than the selected areas shown in Figure 5. (E) Lower magnification two-color lamin protein PALM image registered and overlaid with the corresponding SEM image with a larger field of view than the selected areas shown in Figure 5. (ZIP) [file pone.0077209.s004.zip › Figure-S4/Figure S4D.tif]

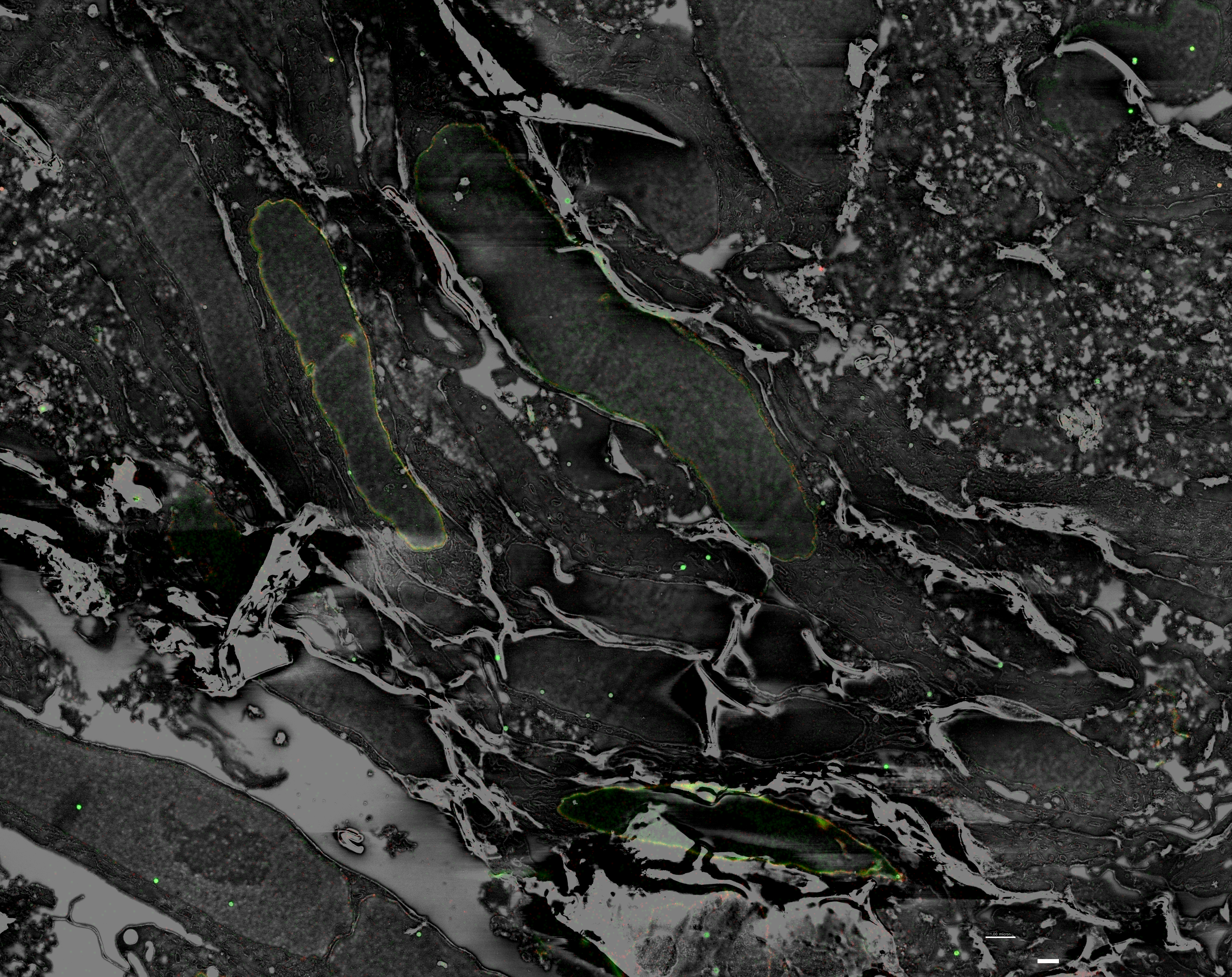

Supplement: Figure S4 — Larger field of view two-color correlative PALM and SEM data of nuclear lamina proteins. (A) Lower magnification PALM image of lamin A-PS-CFP2 with a larger field of view than the selected areas shown in Figure 5. (B) Lower magnification PALM image of lamin B1-mEos2 with a larger field of view than the selected areas shown in Figure 5. (C) Lower magnification of combined lamin A-PS-CFP2 and lamin B1-mEos2 PALM image with a larger field of view than the selected areas shown in Figure 5. (D) Lower magnification SEM image with a larger field of view than the selected areas shown in Figure 5. (E) Lower magnification two-color lamin protein PALM image registered and overlaid with the corresponding SEM image with a larger field of view than the selected areas shown in Figure 5. (ZIP) [file pone.0077209.s004.zip › Figure-S4/Figure S4E.tif]

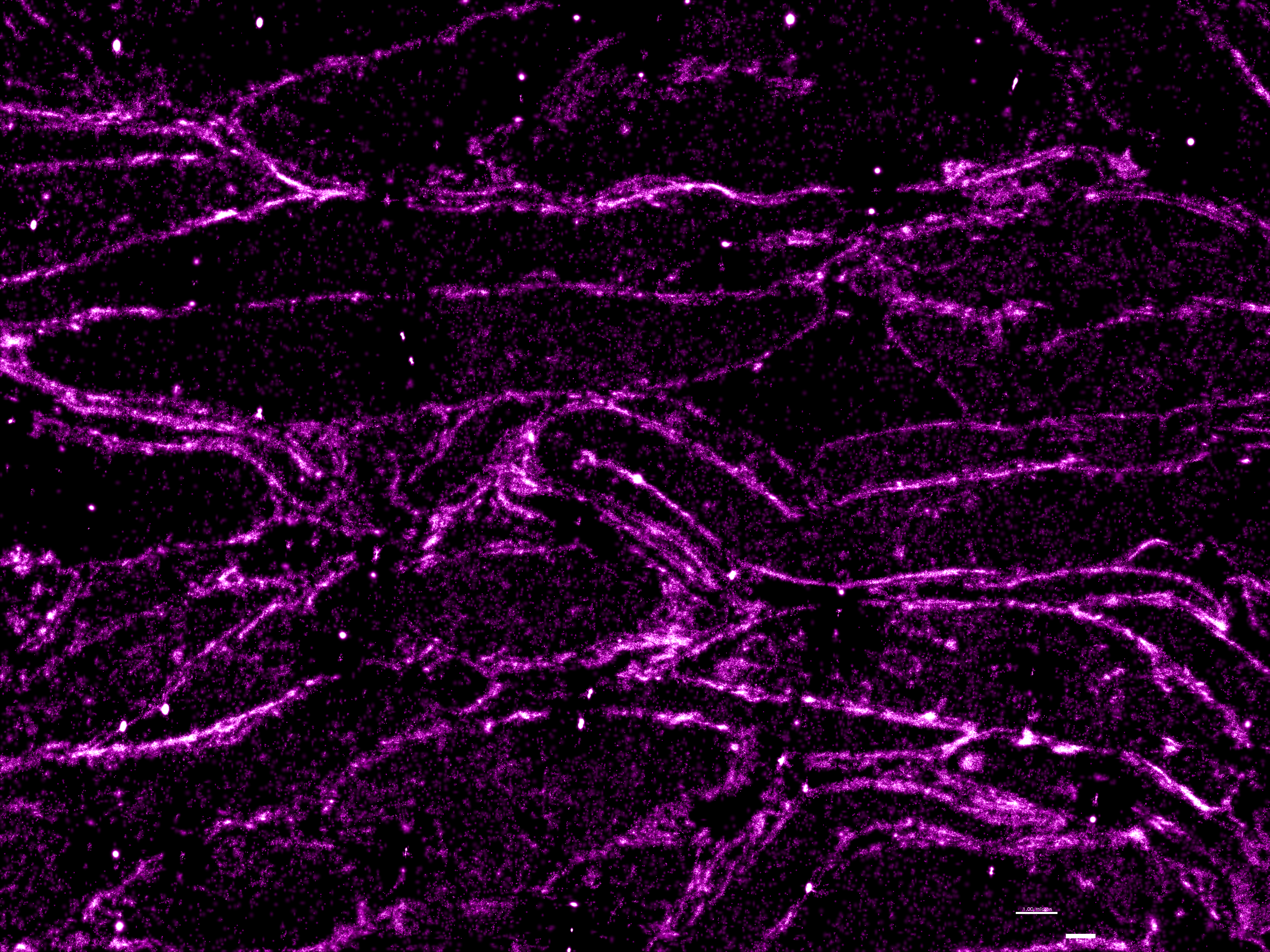

Supplement: Figure S5 — Larger field of view correlated images of caged dye-phalloidin labeled actin PALM data with electron micrographs. (A) Lower magnification PALM image of caged dye labeled actin with a larger field of view than the selected area shown in Figure 6B. (B) Lower magnification SEM image with a larger field of view than the selected area shown in Figure 6C. (C) Lower magnification registered and overlaid PALM and SEM images with a larger field of view than the selected area shown in Figure 6D. (ZIP) [file pone.0077209.s005.zip › Figure-S5/Figure S5A.tif]

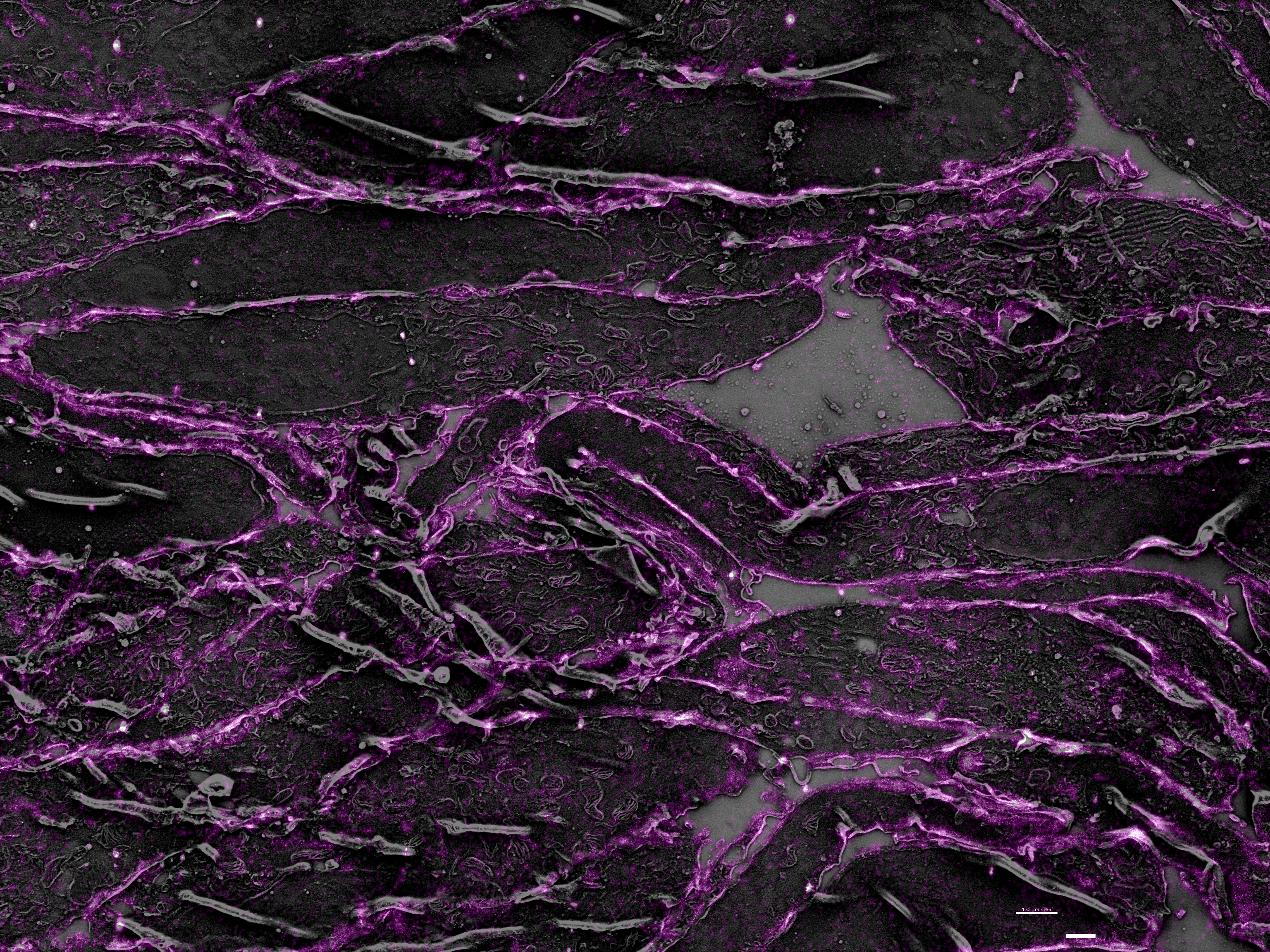

Supplement: Figure S5 — Larger field of view correlated images of caged dye-phalloidin labeled actin PALM data with electron micrographs. (A) Lower magnification PALM image of caged dye labeled actin with a larger field of view than the selected area shown in Figure 6B. (B) Lower magnification SEM image with a larger field of view than the selected area shown in Figure 6C. (C) Lower magnification registered and overlaid PALM and SEM images with a larger field of view than the selected area shown in Figure 6D. (ZIP) [file pone.0077209.s005.zip › Figure-S5/Figure S5C.tif]
